# *PIK3CA* co-occurring mutations and copy-number gain in hormone receptor positive and HER2 negative breast cancer

**DOI:** 10.1038/s41523-022-00382-5

**Published:** 2022-02-18

**Authors:** Ilenia Migliaccio, Marta Paoli, Emanuela Risi, Chiara Biagioni, Laura Biganzoli, Matteo Benelli, Luca Malorni

**Affiliations:** 1 “Sandro Pitigliani” Translational Research Unit, Hospital of Prato, Azienda USL Toscana Centro, 59100 Prato, Italy; 2 Bioinformatics Unit, Hospital of Prato, Azienda USL Toscana Centro, 59100 Prato, Italy; 3 “Sandro Pitigliani” Department of Medical Oncology, Hospital of Prato, Azienda USL Toscana Centro, 59100 Prato, Italy

**Keywords:** Prognostic markers, Cancer genetics, Breast cancer

## Abstract

We aim to elucidate the prognostic value of *PIK3CA* mutations and copy number (CN) gain (*PIK3CA*-mut/gain) in hormone receptor-positive and HER2-negative (HR + /HER2−) breast cancer (BC). We analyzed primary HR + /HER2− BC from three publicly available datasets comprising over 2000 samples and assessed the associations with tumoral and clinical characteristics and outcome. Clinical benefit (CB) in alpelisib-treated patients from two studies including 46 patients was analyzed. About 8–10% of HR + /HER2− primary BC had *PIK3CA*-mut/gain. In two of the datasets analyzed, among patients with *PIK3CA* mutant tumors, those with mut/gain had significantly worse outcome compared to those with CN neutral (*PIK3CA*-mut/neut) and *PIK3CA*-mut/gain remained an independent prognostic factor. CB of alpelisib-treated patients with *PIK3CA*-mut/gain and *PIK3CA*-mut/neut tumors was comparable. *PIK3CA* CN might help clarifying the prognostic and predictive role of *PIK3CA* mutations. Further studies are warranted.

## Introduction

The phosphoinositide 3-kinases (PI3K) pathway plays a critical role in breast cancer (BC) and is frequently altered in hormone receptor positive and HER2 negative (HR + /HER2−) disease^1^. Somatic mutations of the *PIK3CA* gene, encoding for the class IA PI3K p110α subunit, are the most common activating mutations, occurring in 30–50% of ER + /HER2− early BC^2,3^ and in 28% of metastatic disease^4^. Many studies evaluated the prognostic relevance of *PIK3CA* mutations in primary BC with conflicting results^2,5,6^. The approval of alpelisib, a selective PI3K-alpha inhibitor, for the treatment of patients with *PIK3CA* mutant HR + /HER2− advanced BC progressing on prior endocrine therapies^7^ brought to a renewed interest in *PIK3CA* as predictive marker in HR + /HER2− BC.

Gain in *PIK3CA* copy number (CN) has been described in BC^8–18^. It was shown that tumors with high *PIK3CA* CN have more aggressive prognostic features, including large tumor size, high tumor grade, and negative HR status and are more likely to occur in patients with HR and HER2 negative disease^9^. In about half of the tumors, gain in *PIK3CA* CN co-occurs with *PIK3CA* mutations^8,10^.

Despite the overwhelming number of studies assessing the prognostic and predictive role of *PIK3CA* mutations, a comprehensive study combining *PIK3CA* mutations and CN in HR + /HER2− BC is lacking.

In this study, we aimed to perform a combined analysis of *PIK3CA* mutations and CN gain in three large and well characterized BC cohorts, namely METABRIC^19,20^, MSK-breast cancer 2018 (MSK-2018)^21^ and TCGA-BRCA (TCGA)^22^. In addition, we aimed to gain insights on the role of *PIK3CA* gain as a potential predictive marker of response to alpelisib in publicly available datasets of cancer cell lines^23^, patients derived xenograft (PDX)^24^ and patients with metastatic BC^25–27^.

## Results

### *PIK3CA* genomic alterations are associated with tumoral and clinical characteristics in HR + /HER2- BC

**Fig. 1:**
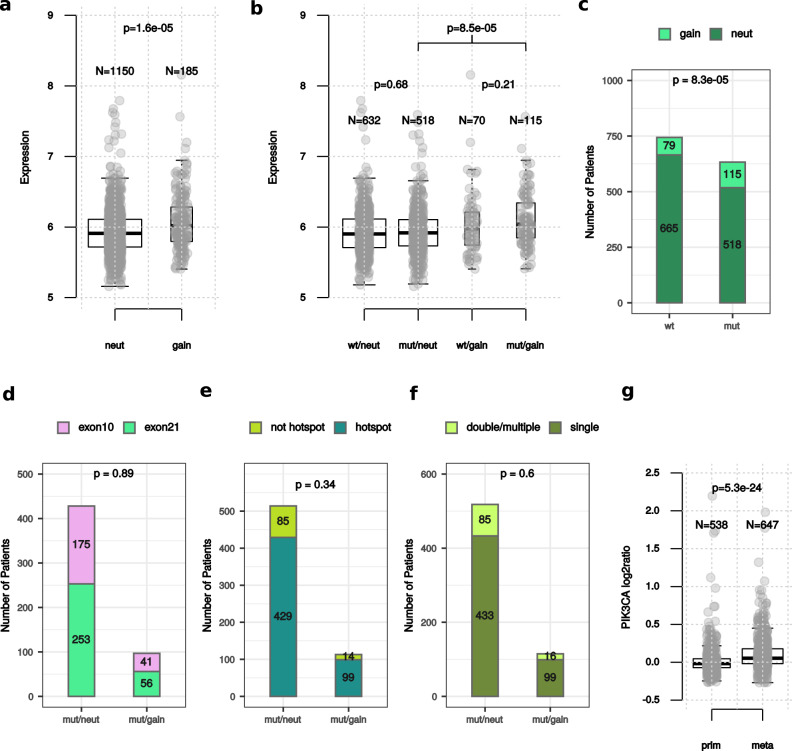
*PIK3CA* gain and *PIK3CA* mRNA or mutational status. Distribution of *PIK3CA* mRNA according to *PIK3CA* categories (**a**, **b**); *PIK3CA* gain according to mutational status (**c**); mutation exons (**d**), hotspots (**e**) and double mutations (**f**) according to *PIK3CA* gain status. Analyses were performed in HR + /HER2− BC within METABRIC using Mann–Whitney–Wilcoxon in **a**, **b** and Two-proportion z-test in **c**–**f**. Levels of *PIK3CA* log2ratio in primary and metastatic HR + /HER2− BC within MSK-2018 (**g**). Mann–Whitney–Wilcoxon test was performed. For box plots, lower and upper bars correspond to the minimum and maximum non-outlier values of the data distribution. Outliers are defined as values outside of the range (Q1 − 1.5×(Q3 − Q1), Q3 + 1.5×(Q3 − Q1)), where Q1 and Q3 are the first and third quartile, respectively.

Gain in *PIK3CA* CN was observed in 194/1377 (14.1%) of HR + /HER2− primary BC within METABRIC. Tumors with gain in *PIK3CA* showed a significant increase in *PIK3CA* mRNA expression compared to *PIK3CA* neutral (*p* = 1.6e−05) (Fig. 1a), even when only *PIK3CA* mutant tumors were considered (*p* = 8.5e−05) (Fig. 1b). Gain in PIK3CA occurred more frequently in *PIK3CA* mutant compared to wild-type (wt) tumors (18.2% versus 10.6% *p* = 8.3e−05) (Fig. 1c). When mutated, tumors with *PIK3CA* gain had a similar proportion of mutations in exons 10 and 21 (*p* = 0.89), hot-spot mutations (p = 0.34) and double mutations (*p* = 0.6) compared to *PIK3CA* neutral (Fig. 1d–f, respectively). These analyses were also performed in luminal A and luminal B BC separately, with similar results (Supplementary Fig. 1).

In MSK-2018, significantly higher levels of *PIK3CA* CN were observed in metastatic HR + /HER2− compared to primary BC samples, both in tumors unselected for *PIK3CA* mutations (*p* = 5.3e−24), and in *PIK3CA* mutant and wt tumors (Fig. 1g and Supplementary Fig. 2). However, the proportion of samples with both *PIK3CA* mutations and CN gain were not significantly different between the de-novo and not de-novo metastatic groups (*p* = 0.17, Supplementary Fig. 2).

Table 1 and Supplementary Tables 1 and 2 show the clinico-pathological characteristics of HR + /HER2− patients within METABRIC, MSK-2018 and TCGA, respectively, according to CN gain and mutational status of *PIK3CA*. Four categories were evaluated: *PIK3CA* wt and CN neutral (-wt/neut), *PIK3CA* wt with CN gain (-wt/gain), *PIK3CA* mutant and CN neutral (-mut/neut), and *PIK3CA* mutant with CN gain (-mut/gain). PIK3CA-mut/gain was observed in 8.3%, 7% and 10% of patients within METABRIC, MSK-2018 and TCGA, respectively. In all datasets *PIK3CA* categories were significantly associated with the histological subtypes. In METABRIC and MSK-2018 a significant association with grade was found. In METABRIC and TCGA significant associations with size and luminal subtypes were also observed. Nodal status and the Integrative Clusters (IC) based on copy number alterations (CNA)^19^ were significantly associated with *PIK3CA* categories in METABRIC. Tumors with *PIK3CA*-gain (both *PIK3CA*-wt/gain and *PIK3CA*-mut/gain) were more frequently of higher grade, larger than 2 cm and luminal B. *PIK3CA*-mut/neut tumors were more frequently lobular or mixed and luminal A. *PIK3CA*-mut/gain was observed with higher frequency within IC 3 and 8, while *PIK3CA*-wt/gain was more frequent in IC 1 and 9.

**Table 1:** PIK3CA categories and clinico-pathological characteristics in ER + /HER2− BC within METABRIC.

		wt/neutral	wt/gain	mut/neutral	mut/gain	*P* value
Age at diagnosis	<50 years	116 (17%)	9 (11%)	85 (16%)	18 (16%)	0.61
	>= 50 years	549 (83%)	70 (89%)	433 (84%)	97 (84%)	
Tumor grade	1	53 (8%)	2 (3%)	96 (19%)	9 (8%)	5e-04
	2	324 (49%)	28 (35%)	264 (51%)	47 (41%)	
	3	251 (38%)	44 (56%)	136 (26%)	57 (50%)	
	NA	37 (6%)	5 (6%)	22 (4%)	2 (2%)	
Tumor size (mm)	<= 20	294 (44%)	32 (41%)	254 (49%)	38 (33%)	0.035
	>20	364 (55%)	47 (59%)	257 (50%)	77 (67%)	
	NA	7 (1%)	0 (0%)	7 (1%)	0 (0%)	
Nodal status	0	342 (51%)	32 (41%)	299 (58%)	64 (56%)	5e−04
	>= 1	290 (44%)	38 (48%)	219 (42%)	51 (44%)	
	NA	33 (5%)	9 (11%)	0 (0%)	0 (0%)	
Histological subtype	Ductal/NST	476 (72%)	64 (81%)	352 (68%)	92 (80%)	0.007
	Lobular	61 (9%)	4 (5%)	45 (9%)	11 (10%)	
	Mixed	80 (12%)	8 (10%)	92 (18%)	10 (9%)	
	Other	31 (5%)	2 (3%)	27 (5%)	1 (1%)	
	NA	17 (3%)	1 (1%)	2 (0%)	1 (1%)	
Molecular subtype	LumA	275 (41%)	27 (34%)	310 (60%)	51 (44%)	5e-04
	LumB	245 (37%)	31 (39%)	109 (21%)	34 (30%)	
	Other	143 (22%)	21 (27%)	96 (19%)	30 (26%)	
	NA	2 (0%)	0 (0%)	3 (1%)	0 (0%)	
Integrative Clusters	1	75 (11%)	15 (19%)	12 (2%)	8 (7%)	5e−04
	2	33 (5%)	2 (3%)	20 (4%)	8 (7%)	
	3	95 (14%)	8 (10%)	155 (30%)	20 (17%)	
	4ER−	8 (1%)	2 (3%)	4 (1%)	1 (1%)	
	4ER +	118 (18%)	6 (8%)	79 (15%)	16 (14%)	
	5	1 (0%)	0 (0%)	1 (0%)	4 (3%)	
	6	58 (9%)	4 (5%)	9 (2%)	6 (5%)	
	7	85 (13%)	6 (8%)	76 (15%)	10 (9%)	
	8	121 (18%)	13 (16%)	131 (25%)	24 (21%)	
	9	57 (9%)	15 (19%)	30 (6%)	12 (10%)	
	10	14 (2%)	8 (10%)	1 (0%)	6 (5%)	

Percentages have been approximated to the nearest whole number

In all datasets we aimed to establish if there were genomic mutations enriched in *PIK3CA*-mut/gain tumors compared to *PIK3CA*-mut/neut and found that *TP53* mutations were indeed significantly enriched (*q* value < 0.05) in primary *PIK3CA*-mut/gain BC samples from METABRIC and MSK-2018 while only a borderline significant association was found in TCGA (*q* value = 0.06). In METABRIC we also found an enrichment of *SF3B1* mutations in *PIK3CA*-mut/gain and of *GATA3* mutations in *PIK3CA*-mut/neut BC. A complete list of the mutations analyzed in all datasets is reported as supplementary table 3.

### *PIK3CA*-mut/gain is significantly and independently associated with outcome in HR + /HER2− BC

**Fig. 2:**
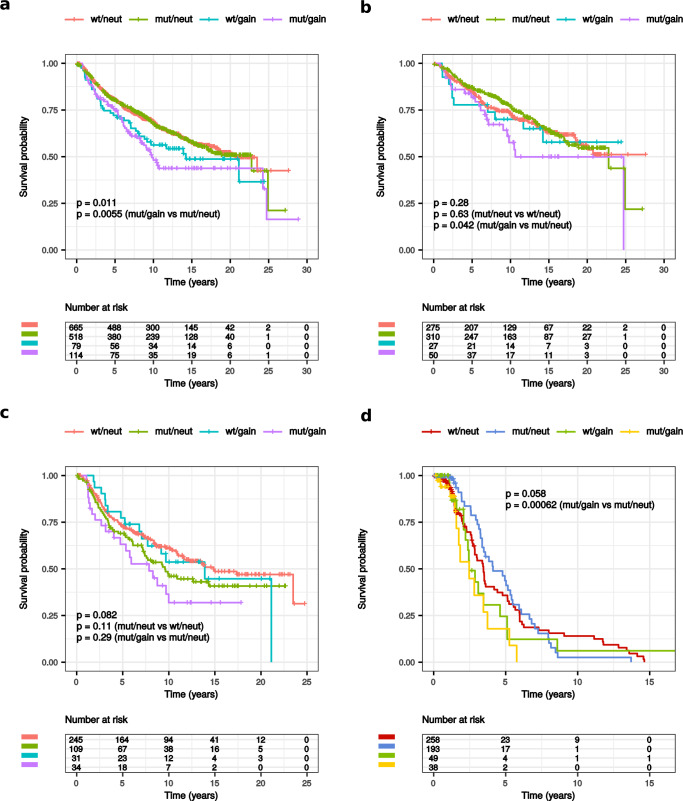
*PIK3CA* categories and survival. Kaplan–Meier curves of RFS according to the four categories of *PIK3CA* in all patients with HR + /HER2− BC (**a**) or in those with luminal A (**b**) or B (**c**) BC within METABRIC and Kaplan–Meier curves of DFS in all patients with primary HR + /HER2− BC within MSK-2018 (**d**). For each category, the number of patients at risk is indicated.

In METABRIC, when comparing the outcome of patients with *PIK3CA*-mut/gain versus those with *PIK3CA*-mut/neut tumors, we found a significantly worse recurrence-free (RFS) (*p* = 0.0055) and disease-specific survival (DSS) (*p* = 0.0026) for the *PIK3CA*-mut/gain group, in both unselected patients and in those with luminal A (RFS *p* = 0.042, DSS *p* = 0.07) but not luminal B BC (RFS *p* = 0.29, DSS *p* = 0.29) (Fig. 2a–c and Supplementary Fig. 3a–c). This was probably related to the different prognostic role of *PIK3CA* mutations observed in patients without *PIK3CA* gain in luminal A versus B subtypes. Indeed, in patients with luminal A BC there was no significant difference in terms of RFS (*p* = 0.63) or DSS (*p* = 0.68) between patients with *PIK3CA*-mut/neut or *PIK3CA*-wt/neut tumors, while in patients with luminal B BC a worse RFS and DSS was found for those with *PIK3CA*-mut/neut compared to *PIK3CA*-wt/neut, even though results were statistically significant only for DSS (*p* = 0.02; RFS *p* = 0.11) (Fig. 2b, c and Supplementary Fig. 3b, c).

In MSK-2018, consistently with data from METABRIC, we observed a significantly worse disease-free survival (DFS) (*p* = 0.00062) for patients with *PIK3CA*-mut/gain compared to *PIK3CA*-mut/neut with overall survival (OS) data only showing a trend for significance (*p* = 0.084) (Fig. 2d and supplementary Fig. 3d).

In TCGA we were unable to confirm the significant association with outcome in patients with HR + /HER2− *PIK3CA*-mut/gain BC (*p* = 0.48), despite the significant associations with poor prognostic factors.

We also analyzed the prognostic value of *PIK3CA* mut/gain in patients with primary HR + /HER2− BC receiving adjuvant endocrine therapy. A significantly worse survival for patients with *PIK3CA*-mut/gain tumors compared to those with *PIK3CA*-mut/neut, in both METABRIC (RFS *p* = 0.0034) and MSK-2018 (DFS *p* = 0.0036) was observed, confirming the poor prognostic role of *PIK3CA*-mut/gain in patients receiving endocrine therapy (supplementary Fig. 4a, b). In addition, we found a higher proportion of *PIK3CA*-mut/gain tumors in patients receiving endocrine therapy who relapsed compared to those who did not relapse (supplementary Fig. 4c).

**Fig. 3:**
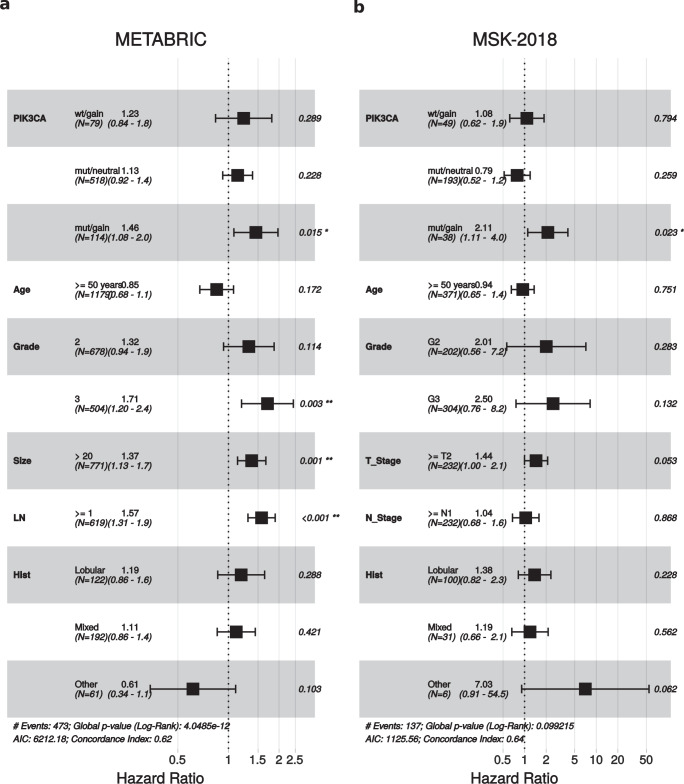
Multivariate analyses. Forest plots showing the results of the Cox multivariate regression analysis for RFS and DFS in METABRIC (**a**) and MSK-2018 (**b**). References were: *PIK3CA* wt/neut, age < 50, grade 1, size < or = 20 mm in **a** and T1 in **b**, N 0 and ductal. The lines represent 95% confidence intervals.

In METABRIC and MSK-2018 we performed multivariate analyses, taking into account age, grade, size, nodal status and histological subtypes and the four categories of *PIK3CA*. *PIK3CA*-mut/gain maintained an independent prognostic role for both RFS (*p* = 0.015) and DSS (*p* = 0.012) in METABRIC, and for DFS (*p* = 0.023) in MSK-2018 (Fig. 3 and supplementary Fig. 5).

### *PIK3CA*-mut/gain does not seem to provide additional informations for alpelisib response in patients with HR + /HER2− BC

**Fig. 4:**
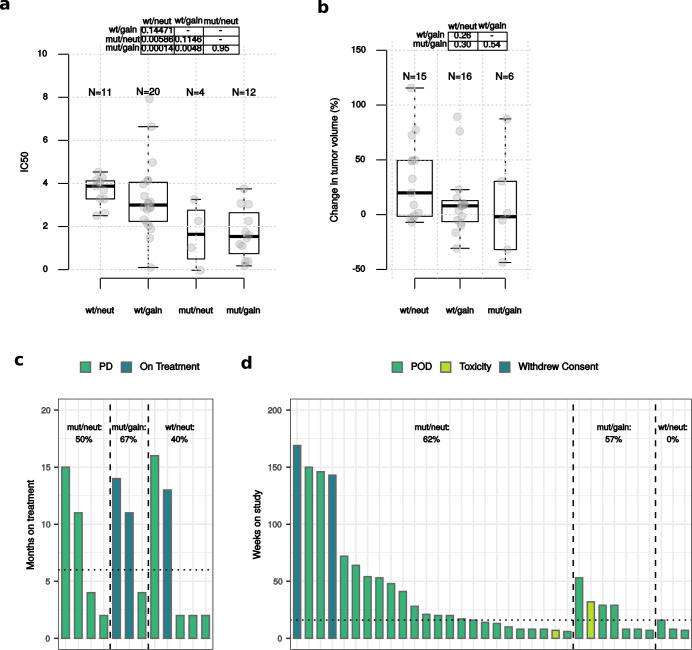
*PIK3CA* categories and response to alpelisib. Box-plots of alpelisib IC50 data in BC cell lines (**a**) and change in tumor volume in BC PDX treated with alpelisib (**b**) according to the *PIK3CA* categories. Bar-plots of month on treatment for alpelisib-treated patients within ALP-2019^26,27^ according to the *PIK3CA* categories (**c**). Bar-plots of weeks on study for alpelisib-treated patients within ALP-2020^25^ according to the *PIK3CA* categories (**d**); Mann–Whitney–Wilcoxon tests were performed in **a** and **b**. For box plots, lower and upper bars correspond to the minimum and maximum non-outlier values of the data distribution. Outliers are defined as values outside of the range (Q1 − 1.5×(Q3 − Q1), Q3 + 1.5×(Q3 − Q1)), where Q1 and Q3 are the first and third quartile, respectively.

We first analyzed cancer cell lines with available IC50 data on alpelisib and *PIK3CA* mutational and CNA data^23^. As expected, both BC and pan-cancer *PIK3CA*-mut/neut cell lines showed significantly lower IC50 values compared to *PIK3CA*-wt/neut (*p* = 0.0059 and *p* = 1.1e−02, respectively) while pan-cancer, but not BC cells with *PIK3CA*-mut/gain showed significantly lower IC50 values compared to *PIK3CA*-mut/neut (*p* = 0.016 and *p* = 0.95, respectively) (Fig. 4a and supplementary Fig. 6a).

We next analyzed the responses to alpelisib in a large and well characterized dataset of PDX^24^. Responses to alpelisib were not significantly different when analyzing only BC PDX (Fig. 4b); however, when considering pan-cancer PDX we observed significantly better responses in tumors with *PIK3CA*-mut/gain compared to *PIK3CA*-mut/neut (*p* = 0.023) (supplementary Fig. 6b).

We finally analyzed patients with ER + /HER2− BC treated with alpelisib and endocrine therapy for metastatic disease included in two different datasets, that were termed ALP-2019^26,27^ and ALP-2020^25^. In ALP-2019, 12 patients received alpelisib and letrozole^26,27^. Among these, three had *PIK3CA*-mut/gain tumors and two of these patients (67%) derived CB; CB rate for patients with *PIK3CA*-mut/neut was 50% (Fig. 4c). In ALP-2020, 34 patients received alpelisib in combination with letrozole or exemestane^25^. Among the 7 patients with *PIK3CA*-mut/gain tumors, 4 (57%) derived CB. CB was observed in 62% of patients with *PIK3CA*-mut/neut tumors (Fig. 4d).

## Discussion

In this study we primarily aimed to perform a comprehensive analysis on the prognostic role of *PIK3CA* CN gain with co-occurring *PIK3CA* mutations in well characterized and publicly available datasets of patients with HR + /HER2− BC.

Previous studies have documented the gain in *PIK3CA* CN in patients with BC, but reports on its frequency have been conflicting, ranging from 1.4%^13^ to as high as 72%^14^, with two of the most recent studies reporting frequencies of 9%^9^ and 17.4%^15^ in HR + and luminal/HER2−, respectively. *PIK3CA* CN has been explored by polymerase chain reaction (PCR)^10–14,16,17^, single nucleotide polymorphism (SNP) array^8,9^ and next generation sequencing (NGS)^15^ and different cut-offs and definitions (*PIK3CA* gain versus amplification) have been used, potentially explaining the wide and discrepant ranges in *PIK3CA* gain frequency. We detected gain in *PIK3CA* CN in 14.1% of HR+/HER2− primary BC in METABRIC, which is in line with two of the most recent reports^9,15^. In the present study we considered together tumors with *PIK3CA* gain and amplification (DNAcopy status 1 and 2, respectively in METABRIC). When analyzing *PIK3CA* mRNA expression in tumors with *PIK3CA* gain and amplification, we found a significantly higher *PIK3CA* mRNA expression for amplified tumors compared to those with gain (*p* = 0.00017), as expected, yet significantly higher mRNA levels were observed in *PIK3CA* gain versus neutral tumors (*p* = 0.02) (supplementary Fig. 7a). Additionally, among patients with *PIK3CA* mutant tumors, a significantly worse RFS was observed for patients with *PIK3CA* amplification or *PIK3CA* gain (supplementary Fig. 7b), indicating that, in patient with *PIK3CA* mutant tumors, *PIK3CA* gain might have clinical relevance in addition to *PIK3CA* amplification.

We observed that *PIK3CA* CN gain occurred preferentially in *PIK3CA* mutant tumors, in accordance with previous reports^8^ and supporting the hypothesis of a potential additive effect of mutations and gain to oncogenesis^8^. Mutations in the helical and kinase domain of *PIK3CA* have been previously associated with different outcome in patients with BC^28^ and double mutations were shown to induce increased PI3K activity and signaling and increased tumor proliferation^29^. In our study, differently from Kadota et al.^8^ we did not find a significant association between gain in *PIK3CA* and any *PIK3CA* mutation exons nor we found any association with *PIK3CA* double mutations or hotspots mutations. Interestingly, we observed a significant increase in *PIK3CA* CN in metastatic compared to primary tumors in MSK-2018, which might suggest a potential role for PIK3CA CN in the metastatic process. However, a correction for cellularity or other confounding factors was not performed, therefore caution must be taken in interpreting this data.

It has been previously demonstrated a significant association between *PIK3CA* CN and high grade, stage and HR- status in an unselected population with BC^9^. Here we demonstrated the significant associations with grade, size and nodal status also in patients with HR+ /HER2− BC. In addition, we found a significant association with luminal subtypes and, accordingly, with the histological subtypes. The significant association with *TP53* mutations is also coherent with these findings.

When we analyzed survival according to the *PIK3CA* categories derived from the combination of CN and mutations, a significantly worse outcome was observed in patients with *PIK3CA*-mut/gain compared to -mut/neut tumors in METABRIC and MSK-2018. Of note, the prognostic role of *PIK3CA*-mut/gain was independent of grade, size, histological subtype and nodal status in both datasets. We also found that *PIK3CA*-mut/gain was prognostic in patients receiving endocrine therapy and that patients relapsing during endocrine therapy had more frequently *PIK3CA*-mut/gain tumors. Whether *PIK3CA*-mut/gain status might be associated with endocrine resistance should be better evaluated in future studies. Prior to our study, the prognostic relevance of *PIK3CA* CN has been demonstrated in pan-cancer studies^30,31^, but in patients with primary HR + BC one of the largest studies failed to establish an association between *PIK3CA* CN and outcome^9^. In previous studies the combined evaluation of *PIK3CA* gain and mutations was not performed. Our results suggest that assessing *PIK3CA* gain together with *PIK3CA* mutations might give a better estimation of the prognostic value of *PIK3CA* in patients with HR + /HER2− BC.

An interesting observation in our study was the different effect on outcome of *PIK3CA* mutations and gain in patients with luminal A and luminal B BC. Compared to patients with *PIK3CA*-mut/neut, those with *PIK3CA*-mut/gain luminal A BC experienced worse RFS. This was not observed in luminal B BC, where patients with *PIK3CA*-mut/neut tumors showed a worse outcome compared to *PIK3CA*-wt/neut. We have not thoroughly investigated the potential explanations of these observations. We analyzed whether *PIK3CA* cancer cell fraction, the DNAcopy status, the presence of double mutations or a different proportion of mutation exons were associated with luminal subtypes. However, no significant differences were found (supplementary Fig. 8). Pereira et al. previously demonstrated that *PIK3CA* mutations have distinct prognostic associations in ER + tumors stratified into IC^20^, and some of these have different proportion of luminal A and luminal B subtypes^19^. Analysing 861 BC samples, Wilson TR et al. showed that patients with *PIK3CA* mutant luminal A BC tended to show a favorable but not statistically significant DFS^32^. This effect was not observed in patients with luminal B BC^32^. It was recently shown that patients with *PIK3CA* mutant luminal A BC were more likely to derive CB from PI3K inhibitors (alpelisib and buparlisib) compared to those with luminal B^26^. Based on ours and previous data it could be hypothesized that *PIK3CA* exerts its effects in a context-dependent manner, but this needs to be tested in future studies. Data regarding the prognostic role of *PIK3CA* mutations in HR +/HER2− BC have been controversial^1,2,5^. Whether different proportion of the luminal subtypes and *PIK3CA* gain might explain the different associations between *PIK3CA* mutations and outcome observed in previous studies remains a hypothesis. Nevertheless, our data support the evaluation of molecular subtypes and *PIK3CA* CN when assessing the prognostic role of *PIK3CA*.

In our study we also aimed to investigate whether a classification based on both *PIK3CA* gain and mutations could help clarifying the predictive role of *PIK3CA* as a marker of alpelisib response. The evidence that double *PIK3CA* mutations results in increased sensitivity to PI3Kα inhibitors compared with single-hotspot mutations^29^ could suggest that multiple hits on *PIK3CA* might have a synergistic effect. In our study better responses to alpelisib were observed in pan-cancer but not BC cell lines and PDX with *PIK3CA*-mut/gain compared to -mut/neut, probably due to the limited sample size. Additionally, patients receiving alpelisib with *PIK3CA*-mut/gain tumors do not seem to show different CB compared to those with *PIK3CA*-mut/neut tumors, which might suggest that response to alpelisib mainly depend upon the *PIK3CA* mutational rather than *PIK3CA* CN status. However, given the limited number of alpelisib-treated patients analyzed in our study, whether *PIK3CA*-mut/gain might predict different sensitivity to PI3K inhibitors needs to be established in larger studies. Also, further studies are needed to clarify if patients with HR +/HER2− tumors with *PIK3CA*-wt/gain might benefit from PI3K inhibitors. Indeed, in BC cells a lower although not statistically significant IC50 for alpelisib was observed, but among patients treated with alpelisib none had *PIK3CA*-wt/gain tumors. Overall, our results encourage the further combined evaluation of *PIK3CA* gain and mutations as a marker of PI3K inhibitors response.

We are aware of the limitations of our study. First, the analyses were retrospective and were performed in very heterogenous populations. Second, comparing CN alteration calls from different datasets is challenging because of the different methodologies and computational approaches used to generate these data. In particular, in METABRIC we used the DNAcopy data, in TCGA we used GISTIC 2.0 data, while in MSK-2018, ALP-2019 and ALP-2020 we utilized log2-ratios data and identified an arbitrary, albeit data-driven, cut-off of *PIK3CA* based on the frequencies observed in METABRIC. We are aware that a univocal and clinically relevant cut-off remains to be set in future studies. For the same reason we did not analyze the enrichment/depletion of CN alterations differentiating *PIK3CA*-mut/gain and mut/neut BC, as done for mutations. Third, data on the predictive role of *PIK3CA*-mut/gain to alpelisib from BC and pan-cancer cells and PDX are not univocal and data from patients treated with alpelisib derive from very limited and non-randomized cohorts. Therefore, results are far to be considered conclusive. Further evidence on the predictive role of PIK3CA-mut/gain is needed from randomized clinical trials. Fourth, experimental approaches are needed to elucidate the mechanisms by which *PIK3CA* gain and *PIK3CA* mutations cooperate in inducing worse outcome and a differential effect in luminal subtypes. As other genes may be co-amplified with *PIK3CA*^33^, it would be interesting to investigate if any of these genes might have a role in the development of an aggressive phenotype in addition to *PIK3CA*.

On the other hand, our data were generated from three large, well characterized cohorts of BC and the poor outcome of patients with *PIK3CA*-mut/gain BC was replicated independently in two of the datasets. We made very interesting and thought-provoking observations: first, patients with HR + /HER2− BC with *PIK3CA*-gain/mut have worse outcome, independently of the most relevant clinico-pathological characteristics; second, *PIK3CA* mutations and CN gain might hold different prognostic effects in luminal A and luminal B BC; third, although very preliminary, our data from pan-cancer cell lines and PDX suggest that response to alpelisib might be influenced by *PIK3CA* CN gain.

In conclusion our data suggest that taking into account *PIK3CA* CN in addition to mutations might bring to a better evaluation of the PI3K pathway and help elucidating some controversial issues regarding the prognostic and predictive role of *PIK3CA*. Given the central role of PI3K pathway in tumor biology, outcome and prediction to therapy in patients with HR + /HER2− BC, further studies evaluating the combined effect of *PIK3CA* gain and mutations are warranted.

## Methods

### Datasets and data collection

For METABRIC^19,20^, genomic, transcriptomic, clinical and outcome data of 2509 primary tumor samples from patients with BC were downloaded from CBioPortal^34,35^ (http://cbioportal.org) and patients with HR + /HER2− BC (*n* = 1413) were selected. *PIK3CA* protein-affecting mutations and CNA based on DNAcopy^36^ were considered. Data on mutated *PIK3CA* exons were downloaded from http://github.com/cclab-brca.

For MSK-2018^21^, genomic, clinical and outcome data of 918 primary and 1000 metastatic tumor samples from 1715 patients with BC were accessed via CBioPortal^34,35^. *PIK3CA* protein-affecting mutations and CNA data based on log2-ratio profiles of HR + /HER2− BC (*n* = 1365) were considered for downstream analyses. Additional clinical data including treatment and de-novo metastatic status were downloaded from the supplementary materials of the original manuscript^21^.

For TCGA^22^, genomic, clinical and outcome data of 1084 primary tumor samples were downloaded from cBioPortal^34,35^. Additional clinical data on patients’ receptor status were downloaded from https://gdc.cancer.gov/access-data/gdc-data-portal, by means of TCGAbiolinks R/Bioconductor package (https://bioconductor.org/packages/release/bioc/html/TCGAbiolinks.html). Patients with HR + /HER2− BC (*n* = 440) were selected, and *PIK3CA* protein-affecting mutations and CNA based on GISTIC 2.0^37^ were considered.

For ALP-2019^26,27^, genomic, clinical and outcome data of 70 primary and metastatic samples from 68 ER + /HER2− patients were downloaded from CBioPortal^34,35^. Based on treatment data (downloaded from the supplementary materials from the original manuscript^26,27^), 12 alpelisib-treated patients were selected for the downstream analysis, along with *PIK3CA* mutational and CNA status based on log2-ratio genomic profiles.

For ALP-2020^25^, genomic, clinical and outcome data for 51 primary and metastatic tumor samples from 51 HR + /HER2− patients treated with alpelisib were downloaded from CBioPortal^34,35^ and *PIK3CA* mutational and CNA status based on log2-ratio were considered for downstream analyses.

For Genomics of Drug Sensitivity in Cancer (GDSC) cell lines^23^, drug data for the PI3K/mTOR inhibitor alpelisib, response data and genetic features of 50 BC and 765 pan-cancer cell lines were downloaded from https://www.cancerrxgene.org/. *PIK3CA* mutational status, CNA data based on GISTIC and drugs IC50 were considered for downstream analyses.

For Novartis Institutes for BioMedical Research (NIBR) PDXE^24^, genomic information, treatment and response data of 277 PDX models across 6 tumor types (BC (*n* = 43), cutaneous melanoma (*n* = 33), colorectal carcinoma (*n* = 59), gastric cancer (*n* = 64), non-small cell lung carcinoma (*n* = 36) and pancreatic ductal adenocarcinoma (*n* = 42)) were retrieved from the original publication^24^. PDX models treated with alpelisib were selected, and *PIK3CA* mutational status, CNA status, treatment and response data were considered for downstream analyses.

### Definition of CNA gain events

For METABRIC, *PIK3CA* CN gains and losses were defined based on DNAcopy calls^36^. Cases with *PIK3CA* CN loss were excluded, leaving 1377 patients for downstream analyses. In MSK-2018, *PIK3CA* CN gains and losses were defined applying the percentile of CN gain and loss events observed in HR + /HER2− patients from METABRIC (0.15 and 0.05 respectively) to the *PIK3CA* log2-ratio values of primary HR + /HER2− samples. As a result, *PIK3CA* log2-ratio greater than 0.1 were considered as CN gain and log2-ratio lower than -0.27 were considered as CN loss events. Cases with *PIK3CA* CN loss were excluded from downstream analysis. The same thresholds were applied to the ALP-2019 and ALP-2020 datasets.

For TCGA *PIK3CA* CN gains and losses were defined based on GISTIC 2.0 calls^37^. After the exclusion of cases with *PIK3CA* CN loss, 413 patients were considered for downstream analyses.

For GDSC cell lines, CN gains and losses were defined based on GISTIC calls. For PDXE, CNA calls from ExomeCNV were used to define PIK3CA gain and loss events.

### Genomic analyses

The lists of the enriched mutations in METABRIC, MSK-2018 and TCGA were generated through cBioPortal^34,35^ by comparing HR + /HER2− BC samples categorized as *PIK3CA*-mut/gain and *PIK3CA*-mut/neut. In MSK-2018, primary and metastatic BC samples were analyzed separately and metastatic samples with unconfirmed HR + /HER2− biopsy status were excluded. As genomic mutations from these datasets were generated using different gene panels, we focused on a list of relevant BC genes from IntOGen (October 2021, *n* = 99)^38^. To make enrichment analyses comparable across the three datasets, statistically significant genes were identified using a restricted hypothesis testing (RHT) analysis. For each dataset, the p-values estimated from the cBioPortal analysis were adjusted by a Benjamini-Hochberg (BH) correction considering the list of relevant BC genes included in the dataset.

### Statistical and survival analyses

Statistical/association analyses, data processing and plots were performed using the R environment (R Core Team, http://cran.r-project.org/). Mann–Whitney–Wilcoxon test was used to check for significant shifts between two distributions. Two-proportion z-test was used to compare proportions in two groups. Fisher’s exact test was used for comparison between two categorical variables. Tests were performed two-sided. Kaplan–Meier curves and log-rank test were used for all survival analyses. Cox proportional hazards model was used for multivariate analyses, including as covariates *PIK3CA* status, age, tumor grade, tumor size, histological subtypes and lymph-node status.

For METABRIC and MSK-2018 clinical endpoints were retrieved from cBioPortal: DSS and RFS for METABRIC, OS and DFS for MSK-2018. For ALP-2019, patients were considered to achieve CB when showed a stable disease for more than 6 months, in accordance with^26^; while for ALP-2020, patients were considered to achieve CB when showed a stable disease for more than 16 weeks, as in^25^. For GDSC cell lines, drug-specific IC50 values were downloaded from https://www.cancerrxgene.org/. For NIBR PDXE, best average response to alpelisib (change in tumor volume %) was considered^24^.

Within MSK-2018, patients with synchronous primary tumors and de novo metastatic were excluded from analyses involving primary samples; to avoid duplicates, when more than one sample was present, the treatment naïve one was chosen. When duplicated samples could not be solved, cases were excluded. For survival analysis in patients treated with endocrine therapy, patients with primary HR + /HER2− BC treated with hormonal adjuvant systemic therapy were selected.

### Reporting summary

Further information on research design is available in the Nature Research Reporting Summary linked to this article.

## Supplementary information

supplementary information

Reporting Summary

Supplemental Material File #1

## Data Availability

This study collected and analyzed data deriving from publicly available datasets^19–26^.

## Code availability

Codes used to generate the data are available upon reasonable request.

## References

[1] Criscitiello C, Marra A, Curigliano G (2021). PIK3CA mutation assessment in HR+/HER2− metastatic breast cancer: overview for oncology clinical practice. J. Mol. Pathol..

[2] Zardavas D (2018). Tumor PIK3CA genotype and prognosis in early-stage breast cancer: a pooled analysis of individual patient data. J. Clin. Oncol..

[3] Luen SJ (2018). Association of somatic driver alterations with prognosis in postmenopausal, hormone receptor-positive, HER2-negative early breast cancer: a secondary analysis of the BIG 1-98 randomized clinical trial. JAMA Oncol..

[4] Mosele F (2020). Outcome and molecular landscape of patients with PIK3CA-mutated metastatic breast cancer. Ann. Oncol..

[5] Pang, B. et al. Prognostic role of PIK3CA mutations and their association with hormone receptor expression in breast cancer: a meta-analysis. *Sci. Rep.* **4** (2014). 10.1038/srep06255 PMC4150110 25176561

[6] Sobhani N (2018). The prognostic value of PI3K mutational status in breast cancer: a meta-analysis. J. Cell. Biochem..

[7] André F (2019). Alpelisib for PIK3CA-mutated, hormone receptor–positive advanced breast cancer. N. Engl. J. Med..

[8] Kadota M (2009). Identification of novel gene amplifications in breast cancer and coexistence of gene amplification with an activating mutation of PIK3CA. Cancer Res..

[9] Gonzalez-Angulo AM (2013). Frequency of mesenchymal-epithelial transition factor gene (MET) and the catalytic subunit of phosphoinositide-3-kinase (PIK3CA) copy number elevation and correlation with outcome in patients with early stage breast cancer. Cancer.

[10] Wu, G. et al. Somatic mutation and gain of copy number of PIK3CA in human breast cancer. *Breast Cancer Res.* **7**, (2005). 10.1186/bcr1262 PMC1242128 16168105

[11] Lee MH, Cho JH, Kwon SY, Jung SJ, Lee JH (2020). Clinicopathological characteristics of PIK3CA mutation and amplification in Korean patients with breast cancers. Int. J. Med. Sci..

[12] Hosseini S (2018). Relationship between PIK3CA amplification and P110α and CD34 tissue expression as angiogenesis markers in Iranian women with sporadic breast cancer. Iran. J. Pathol..

[13] Campbell IG (2004). Mutation of the PIK3CA gene in ovarian and breast cancer. Cancer Res..

[14] Firoozinia M, Jahromi MZ, Moghadamtousi SZ, Nikzad S, Kadir HA (2014). PIK3CA gene amplification and PI3K p110α protein expression in breast carcinoma. Int. J. Med. Sci..

[15] Loibl S (2019). Mutational diversity and therapy response in breast cancer: a sequencing analysis in the neoadjuvant geparsepto trial. Clin. Cancer Res..

[16] López-Knowles E (2010). PI3K pathway activation in breast cancer is associated with the basal-like phenotype and cancer-specific mortality. Int. J. Cancer.

[17] Fumagalli D (2016). Somatic mutation, copy number and transcriptomic profiles of primary and matched metastatic estrogen receptor-positive breast cancers. Ann. Oncol..

[18] Chia SKL (2019). PIK3CA alterations and benefit with neratinib: analysis from the randomized, double-blind, placebo-controlled, phase III ExteNET trial. Breast Cancer Res..

[19] Curtis C (2012). The genomic and transcriptomic architecture of 2,000 breast tumours reveals novel subgroups. Nature.

[20] Pereira, B. et al. The somatic mutation profiles of 2,433 breast cancers refines their genomic and transcriptomic landscapes. *Nat. Commun.* **7**, (2016). 10.1038/ncomms11479 PMC4866047 27161491

[21] Razavi P (2018). The genomic landscape of endocrine-resistant advanced breast cancers. Cancer Cell.

[22] Hoadley KA (2018). Cell-of-origin patterns dominate the molecular classification of 10,000 tumors from 33 types of cancer. Cell.

[23] Yang W (2013). Genomics of Drug Sensitivity in Cancer (GDSC): a resource for therapeutic biomarker discovery in cancer cells. Nucleic Acids Res..

[24] Gao H (2015). High-throughput screening using patient-derived tumor xenografts to predict clinical trial drug response. Nat. Med..

[25] Razavi P (2020). Alterations in PTEN and ESR1 promote clinical resistance to alpelisib plus aromatase inhibitors. Nat. Cancer.

[26] Nixon, M. J. et al. PIK3CA and MAP3K1 alterations imply luminal A status and are associated with clinical benefit from pan-PI3K inhibitor buparlisib and letrozole in ER+ metastatic breast cancer. *npj Breast Cancer* **5** (2019). 10.1038/s41523-019-0126-6 PMC6757060 31552290

[27] Mayer IA (2017). A phase Ib study of alpelisib (BYL719), a PI3Kα-specific inhibitor, with letrozole in ER+/HER2- metastatic breast cancer. Clin. Cancer Res..

[28] Barbareschi M (2007). Different prognostic roles of mutations in the helical and kinase domains of the PIK3CA gene in breast carcinomas. Clin. Cancer Res..

[29] Vasan N (2019). Double PIK3CA mutations in cis increase oncogenicity and sensitivity to PI3Ka inhibitors. Science.

[30] Zhang Y (2017). A Pan-cancer proteogenomic atlas of PI3K/AKT/mTOR pathway alterations. Cancer Cell.

[31] Smith, J. C. & Sheltzer, J. M. Systematic identification of mutations and copy number alterations associated with cancer patient prognosis. *eLife* **7** (2018). 10.7554/eLife.39217 PMC6289580 30526857

[32] Wilson TR (2016). The molecular landscape of high-risk early breast cancer: comprehensive biomarker analysis of a phase III adjuvant population. npj Breast Cancer.

[33] Boberg DR (2013). Copy number variation in ACHE/EPHB4 (7q22) and in BCHE/MME (3q26) genes in sporadic breast cancer. Chem. Biol. Interact..

[34] Cerami E (2012). The cBio Cancer Genomics Portal: an open platform for exploring multidimensional cancer genomics data. Cancer Discov..

[35] Gao, J. et al. Integrative analysis of complex cancer genomics and clinical profiles using the cBioPortal. *Sci. Signal.* **6** (2013). 10.1126/scisignal.2004088 PMC4160307 23550210

[36] Olshen AB, Venkatraman ES, Lucito R, Wigler M (2004). Circular binary segmentation for the analysis of array-based DNA copy number data. Biostatistics.

[37] Mermel CH (2011). GISTIC2.0 facilitates sensitive and confident localization of the targets of focal somatic copy-number alteration in human cancers. Genome Biol..

[38] Martínez-Jiménez F (2020). A compendium of mutational cancer driver genes. Nat. Rev. Cancer.

