# Supplementary material for: PIK3CA co-occurring mutations and copy-number gain in hormone receptor positive and HER2 negative breast cancer
Source: NPJ Breast Cancer. 2022 Feb 18;8:24. doi: 10.1038/s41523-022-00382-5 (PMC8857304; doi:10.1038/s41523-022-00382-5)
Supplement: Supplementary file 1 — supplementary information [file 41523_2022_382_MOESM1_ESM.pdf]

Co-occurring mutations and copy number gain in *PIK3CA* define a distinct group with poor prognosis among patients with hormone receptor positive and HER2 negative breast cancer

Ilenia Migliaccio, Marta Paoli, Emanuela Risi, Chiara Biagioni, Laura Biganzoli, Matteo Benelli and Luca Malorni

Hospital of Prato

Supplementary information

Supplementary Figure 1: Distribution of *PIK3CA* mRNA expression according to *PIK3CA* categories (a-b); *PIK3CA* gain according to mutational status (c-d); *PIK3CA* mutation exons (e-f), hot spot (g-h) and double mutations (i-l) according to gain. Analyses were performed in HR+/ HER2- luminal A (a-c-e-g-i) and luminal B (b-d-f-h-l) BC within METABRIC using Mann-Whitney-Wilcoxon in (a) (b) and Two-proportion z-test in (c) (d) (e) (f) (g) (h) (i) (l). For box plots, lower and upper bars correspond to the minimum and maximum non-outlier values of the data distribution. Outliers are defined as values outside of the range (Q1 − 1.5 × (Q3 − Q1), Q3 + 1.5 × (Q3 − Q1)), where Q1 and Q3 are the first and third quartile, respectively.

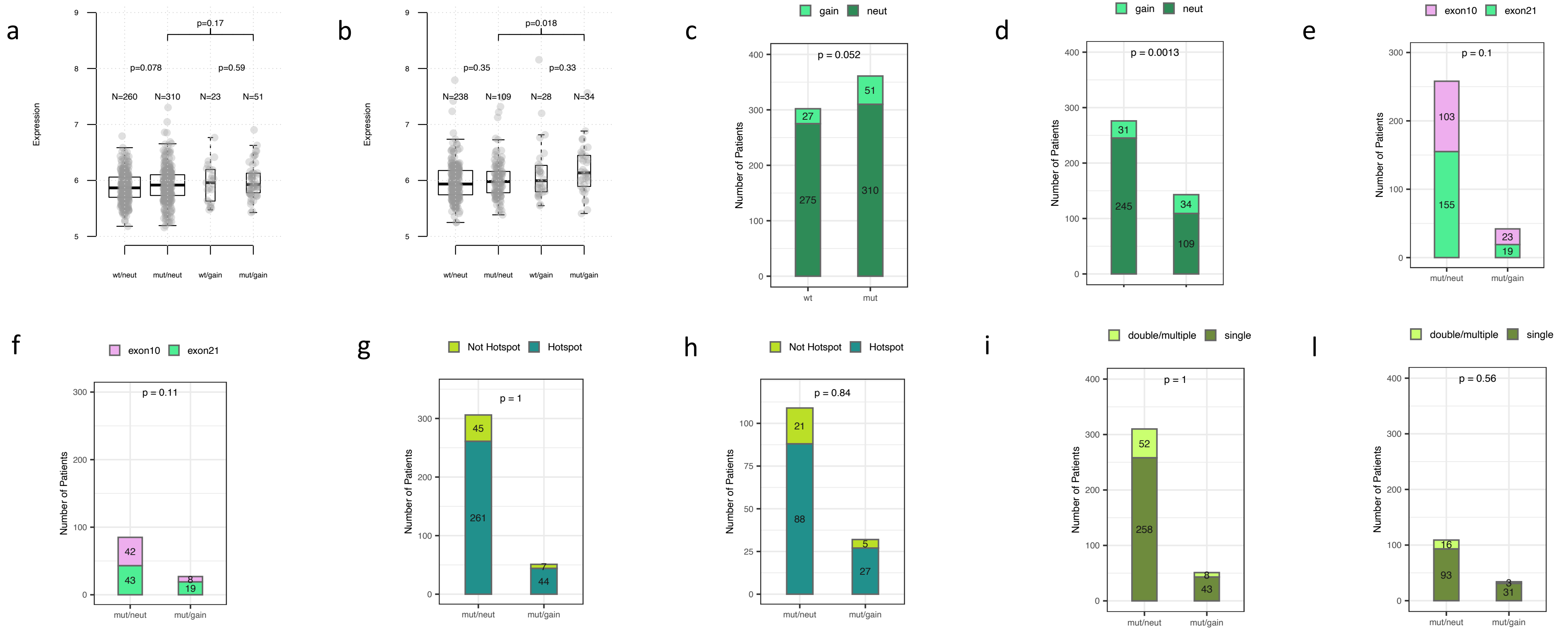

Migliaccio I et al.

Supplementary Figure 2: Levels of *PIK3CA* log2ratio in primary and metastatic HR+/HER2- *PIK3CA* wt (a) and mutant (b) BC within MSK-2018. Mann-Whitney-Wilcoxon test was performed. Distribution of the *PIK3CA* categories in de-novo and not de-novo metastatic samples within MSK-2018 (c). For box plots, lower and upper bars correspond to the minimum and maximum non-outlier values of the data distribution. Outliers are defined as values outside of the range (Q1 − 1.5 × (Q3 − Q1), Q3 + 1.5 × (Q3 − Q1)), where Q1 and Q3 are the first and third quartile, respectively.

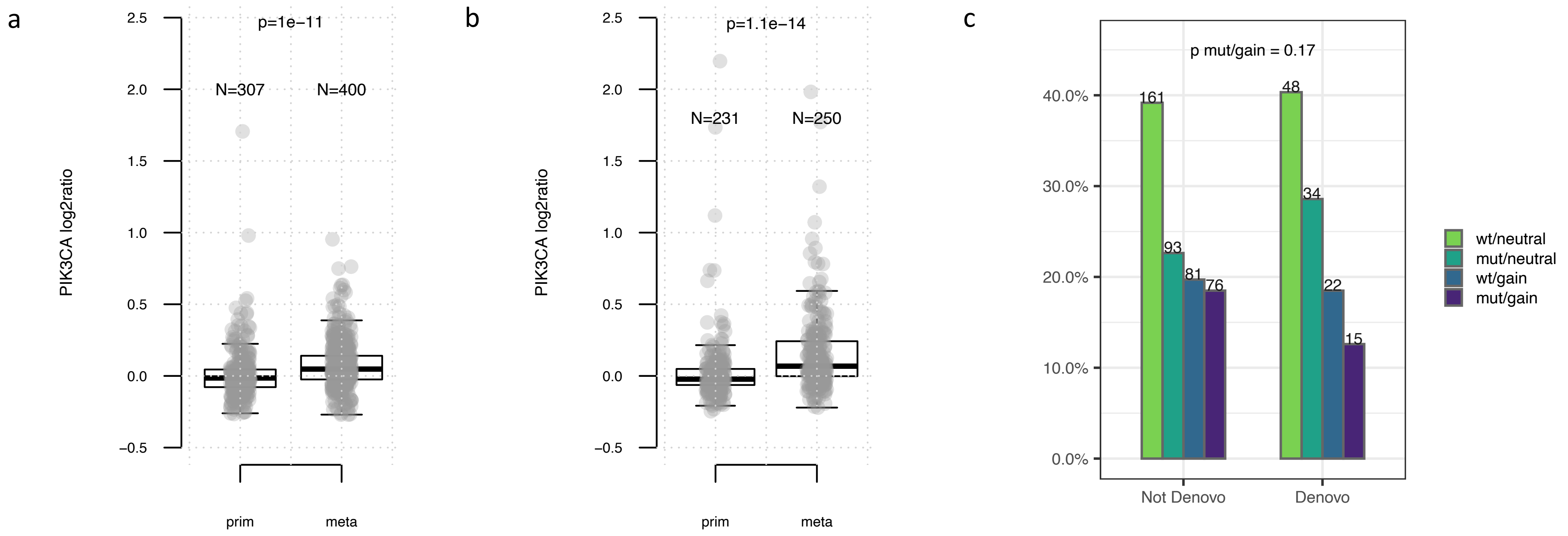

Migliaccio I et al.

Supplementary table 1: Distribution of PIK3CA categories according to clinico-pathological characteristics in patients with HR+/HER2- BC within MSK-2018

		wt/neutral	wt/gain	mut/neutral	mut/gain	P value
**Invasive Carcinoma Diagnosis Age**	< 50 years	95 (37%)	22 (45%)	58 (30%)	10 (26%)	0.13
	>= 50 years	163 (63%)	27 (55%)	135 (70%)	28 (74%)	
**Menopausal Status At Diagnosis**	Pre	101 (39%)	21 (43%)	65 (34%)	10 (26%)	0.2
	Post	134 (52%)	26 (53%)	112 (58%)	27 (71%)	
	Other	21 (8%)	1 (2%)	16 (8%)	1 (3%)	
	NA	2 (1%)	1 (2%)	0 (0%)	0 (0%)	
**Overall Primary Tumor Grade**	G1	14 (5%)	1 (2%)	33 (17%)	1 (3%)	5e-04
	G2	89 (34%)	4 (8%)	85 (44%)	12 (32%)	
	G3	147 (57%)	44 (90%)	70 (36%)	21 (55%)	
	NA	8 (3%)	0 (0%)	5 (3%)	4 (11%)	
**T Stage**	T1	151 (59%)	25 (51%)	128 (66%)	23 (61%)	0.17
	>= T2	107 (41%)	24 (49%)	65 (34%)	15 (39%)	
**N Stage**	N0	153 (59%)	25 (51%)	122 (63%)	24 (63%)	0.45
	>= N1	105 (41%)	24 (49%)	71 (37%)	14 (37%)	
**Tumor Sample Histology**	Ductal	204 (79%)	43 (88%)	127 (66%)	33 (87%)	0.009
	Lobular	38 (15%)	3 (6%)	50 (26%)	5 (13%)	
	Mixed	12 (5%)	3 (6%)	14 (7%)	0 (0%)	
	Other	4 (2%)	0 (0%)	2 (1%)	0 (0%)	

Supplementary table 2: Distribution of *PIK3CA* categories according to clinico-pathological characteristics in patients with HR+/HER2- BC within TCGA

		wt/neutral	wt/gain	mut/neutral	mut/gain	P value
**Age**	< 50 years	50 (26%)	13 (19%)	29 (26%)	9 (21%)	0.56
	>= 50 years	139 (74%)	57 (81%)	83 (74%)	33 (79%)	
**T Stage**	T1	55 (29%)	12 (17%)	41 (37%)	10 (24%)	0.026
	>= T2	134 (71%)	57 (81%)	71 (63%)	32 (76%)	
	NA	0 (0%)	1 (1%)	0 (0%)	0 (0%)	
**N Stage**	N0	90 (48%)	30 (43%)	55 (49%)	24 (57%)	0.28
	>= N1	97 (51%)	37 (53%)	57 (51%)	18 (43%)	
	NA	2 (1%)	3 (4%)	0 (0%)	0 (0%)	
**Tumor Sample Histology**	Ductal	104 (55%)	61 (87%)	69 (62%)	35 (83%)	5e-04
	Lobular	58 (31%)	5 (7%)	32 (29%)	2 (5%)	
	Mixed (NOS)	6 (3%)	1 (1%)	4 (4%)	2 (5%)	
	Other	21 (11%)	3 (4%)	7 (6%)	3 (7%)	
PAM50	LumA	109 (58%)	31 (44%)	94 (84%)	25 (60%)	5e-04
	LumB	37 (20%)	22 (31%)	13 (12%)	13 (31%)	
	Other	11 (6%)	13 (19%)	4 (4%)	3 (7%)	
	NA	32 (17%)	4 (6%)	1 (1%)	1 (2%)	

Supplementary Figure 3: Kaplan–Meier curves of DSS according to the four categories of *PIK3CA* in all patients with HR+/HER2- BC (a) or in those with luminal A (b) or B (c) tumors within METABRIC and of OS in all patients with primary HR+/HER2- BC within MSK-2018 (d). For each category, the number of patients at risk is indicated.

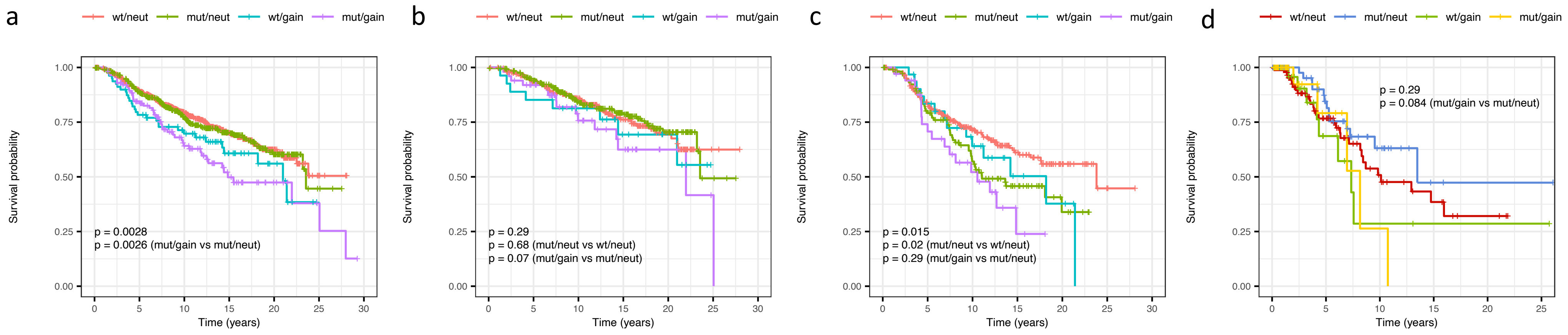

a — Number at risk

Time (years)	0	5	10	15	20	25	30
wt/neut	665	542	349	191	61	4	0
mut/neut	518	420	273	159	55	3	0
wt/gain	79	61	44	20	8	0	0
mut/gain	114	85	48	22	8	3	0

b — Number at risk

Time (years)	0	5	10	15	20	25	30
wt/neut	275	230	157	88	30	2	0
mut/neut	310	268	180	107	39	3	0
wt/gain	27	23	17	9	5	0	0
mut/gain	50	42	23	13	4	1	0

c — Number at risk

Time (years)	0	5	10	15	20	25	30
wt/neut	245	187	110	57	21	2	0
mut/neut	109	77	46	20	5	0	0
wt/gain	31	25	15	5	3	0	0
mut/gain	34	21	12	2	0	0	0

d — Number at risk

Time (years)	0	5	10	15	20	25
wt/neut	258	42	16	8	3	0
mut/neut	193	28	11	2	1	1
wt/gain	49	7	2	1	1	1
mut/gain	38	5	1	0	0	0

Migliaccio I et al.

Supplementary Figure 4: Kaplan–Meier curves of RFS according to the four categories of *PIK3CA* in patients with HR+/HER2- BC treated with endocrine therapies within METABRIC (a) and of DFS in patients with primary HR+/HER2- BC treated with endocrine therapies within MSK-2018 (b). For each category, the number of patients at risk is indicated. Distribution of the *PIK3CA* categories in relapsed and not-relapsed endocrine-treated patients within METABRIC (c).

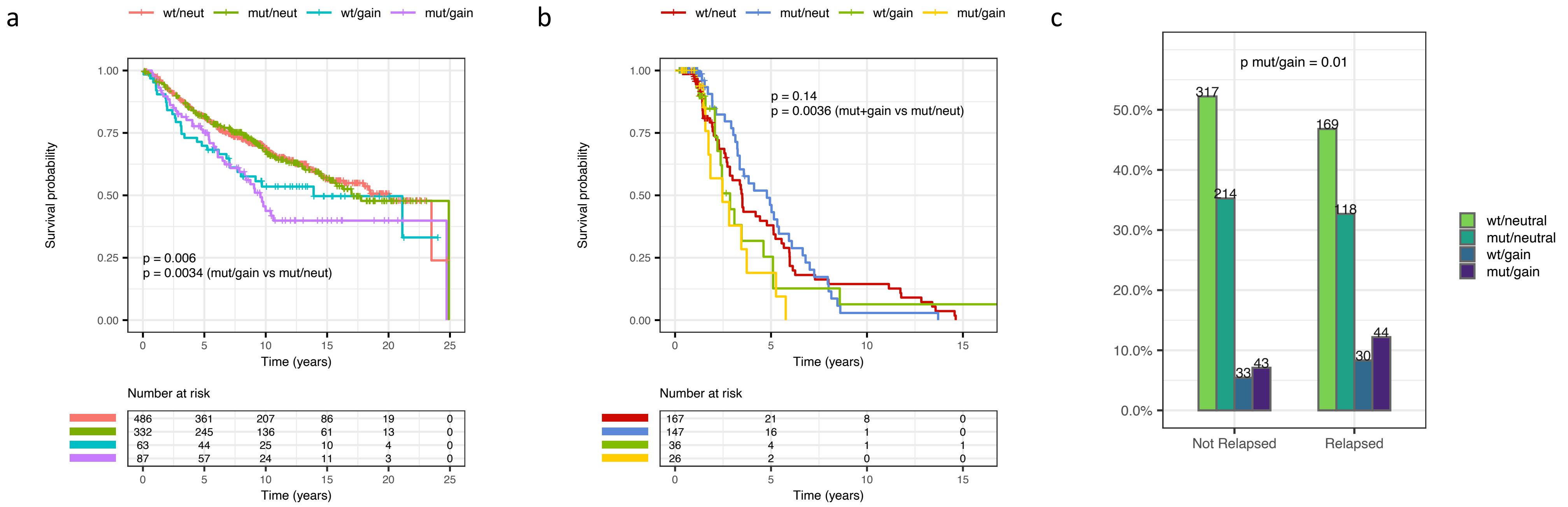

Migliaccio I et al.

Supplementary figure 5: Forest plots showing the results of the multivariate regression analysis for DSS in METABRIC (a) and OS in MSK-2018 (b).

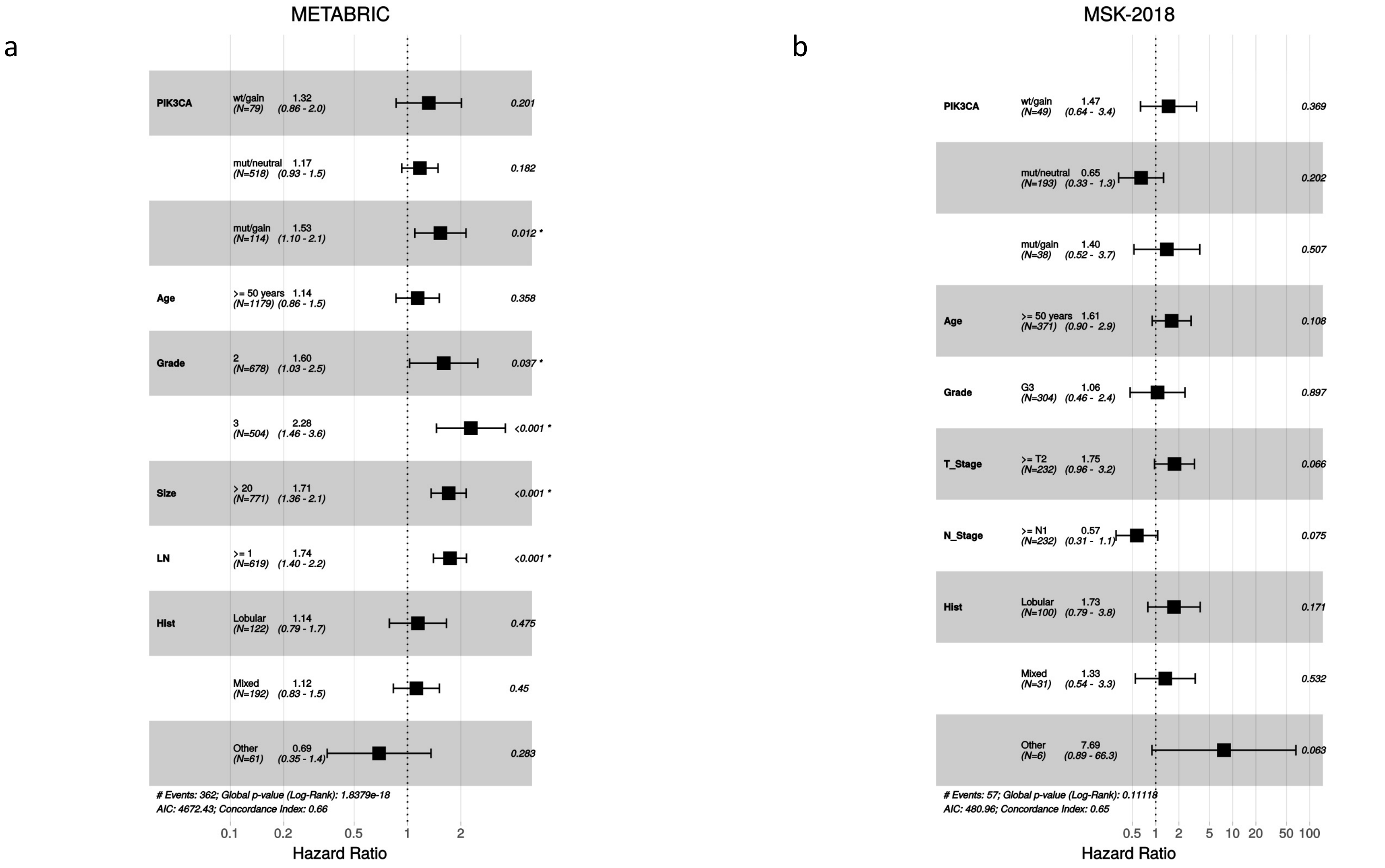

Migliaccio I et al.

Supplementary Figure 6: Box-plots of alpelisib IC50 data in pan-cancer cell lines according to the *PIK3CA* categories (a). Change in tumor volume in pan-cancer PDX treated with alpelisib according to the *PIK3CA* categories (b). Lower and upper bars correspond to the minimum and maximum non-outlier values of the data distribution. Outliers are defined as values outside of the range (Q1 − 1.5 × (Q3 − Q1), Q3 + 1.5 × (Q3 − Q1)), where Q1 and Q3 are the first and third quartile, respectively.

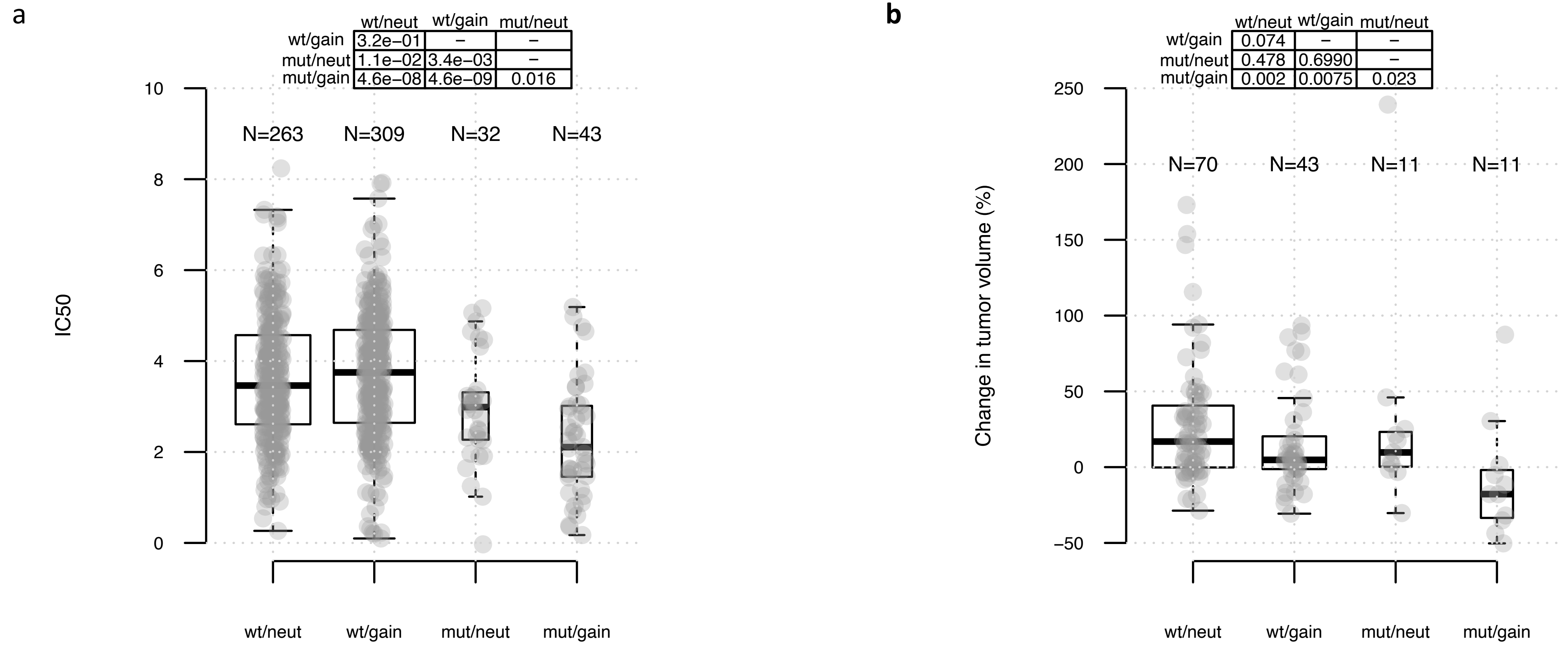

Migliaccio I et al.

Supplementary Figure 7: Distribution of *PIK3CA* mRNA expression in tumors with *PIK3CA* neutral, gain or amplified DNAcopy status (a). Kaplan-Meier curves of RFS for all patients with HR+/HER2- *PIK3CA* mutant BC according to *PIK3CA* neutral, gain or amplified DNAcopy status. For box plot, lower and upper bars correspond to the minimum and maximum non-outlier values of the data distribution. Outliers are defined as values outside of the range (Q1 − 1.5 × (Q3 − Q1), Q3 + 1.5 × (Q3 − Q1)), where Q1 and Q3 are the first and third quartile, respectively.

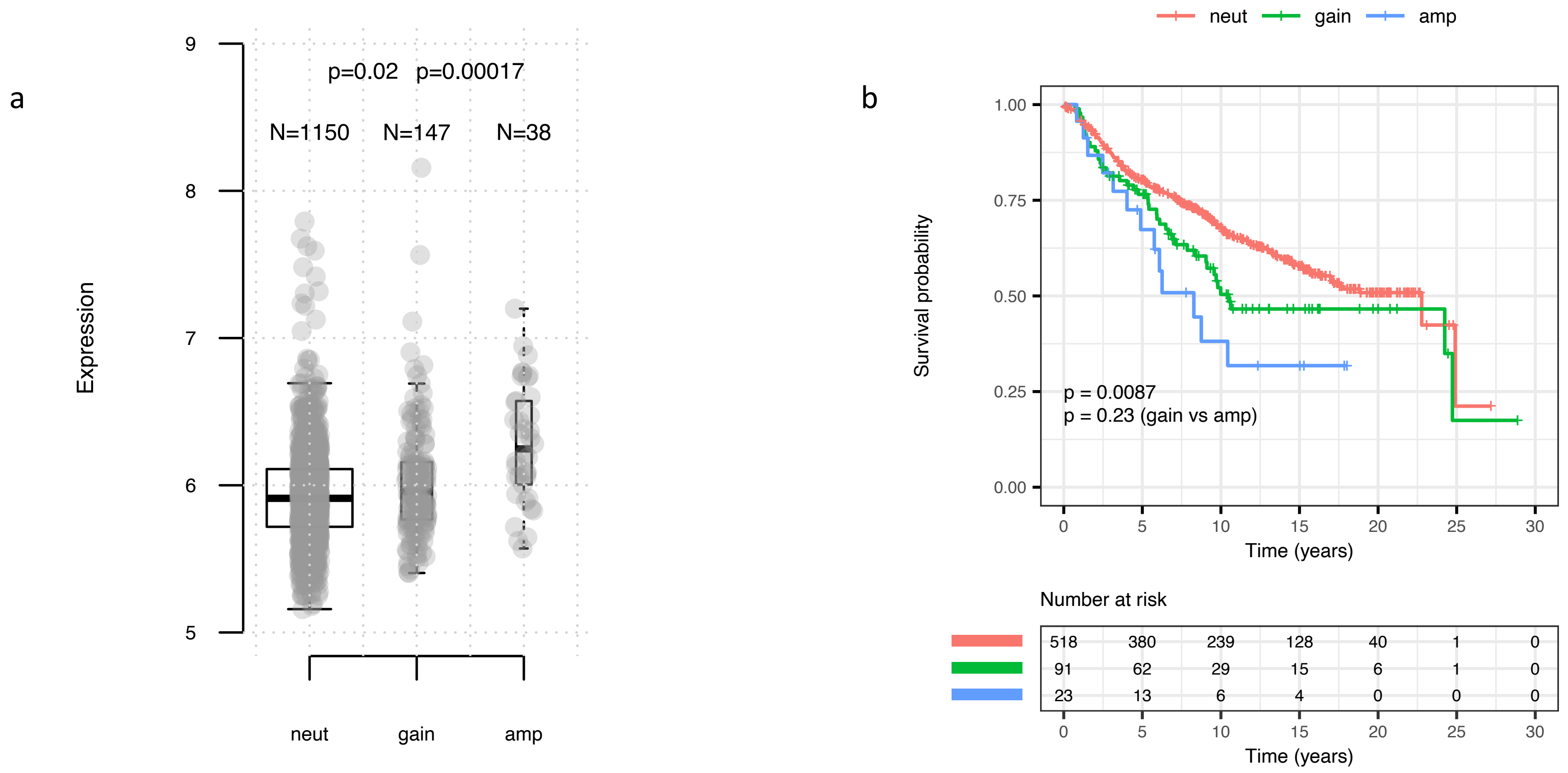

Migliaccio I et al.

Supplementary Figure 8: *PIK3CA* cancer cell fraction (a), DNAcopy status (b), double mutations (c) and mutation exons (d) according to luminal status in HR+/HER2- BC within METABRIC. For box plots, lower and upper bars correspond to the minimum and maximum non-outlier values of the data distribution. Outliers are defined as values outside of the range (Q1 − 1.5 × (Q3 − Q1), Q3 + 1.5 × (Q3 − Q1)), where Q1 and Q3 are the first and third quartile, respectively.

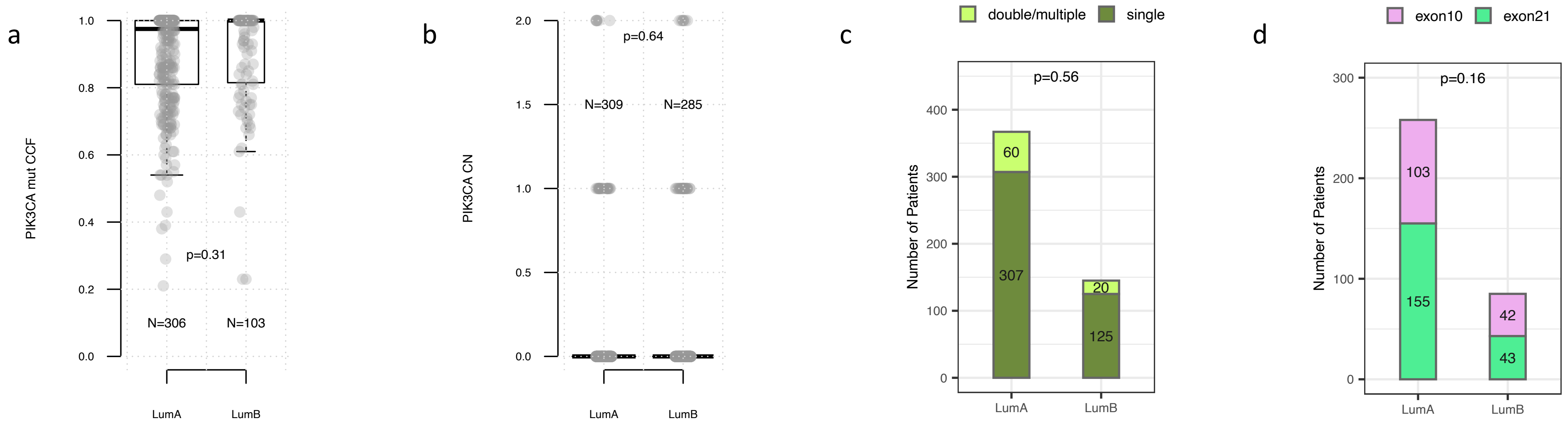

Supplemetary table 3

dataset	Gene	Cytoband	X.A..mut.gain	X.B..mut.neut	Log.Ratio	p.Value	q.Value	Enriched.in	type
TCGA	TP53	17p13.1	15 (36.59%)	13 (11.50%)	1.67	0.0007132	0.064188	(A) mut/gain	mut
TCGA	CDH1	16q22.1	2 (4.88%)	30 (26.55%)	-2.44	0.001679	0.075555	(B) mut/neut	mut
TCGA	KMT2C	7q36.1	3 (7.32%)	22 (19.47%)	-1.41	0.0534	0.742247191011236	(B) mut/neut	mut
TCGA	MYH11	16p13.11	3 (7.32%)	1 (0.88%)	3.05	0.058	0.742247191011236	(A) mut/gain	mut
TCGA	TBX3	12q24.21	0 (0.00%)	8 (7.08%)	<-10	0.0785	0.742247191011236	(B) mut/neut	mut
TCGA	LRP1B	2q22.1-q22.2	0 (0.00%)	7 (6.19%)	<-10	0.109	0.742247191011236	(B) mut/neut	mut
TCGA	ERBB2	17q12	0 (0.00%)	6 (5.31%)	<-10	0.151	0.742247191011236	(B) mut/neut	mut
TCGA	FAT1	4q35.2	0 (0.00%)	6 (5.31%)	<-10	0.151	0.742247191011236	(B) mut/neut	mut
TCGA	FAT3	11q14.3	0 (0.00%)	6 (5.31%)	<-10	0.151	0.742247191011236	(B) mut/neut	mut
TCGA	KMT2D	12q13.12	0 (0.00%)	6 (5.31%)	<-10	0.151	0.742247191011236	(B) mut/neut	mut
TCGA	MYH9	22q12.3	0 (0.00%)	6 (5.31%)	<-10	0.151	0.742247191011236	(B) mut/neut	mut
TCGA	NOTCH2	1p12	0 (0.00%)	6 (5.31%)	<-10	0.151	0.742247191011236	(B) mut/neut	mut
TCGA	AFF3	2q11.2	2 (4.88%)	1 (0.88%)	2.46	0.173	0.742247191011236	(A) mut/gain	mut
TCGA	NCOR1	17p12-p11.2	4 (9.76%)	5 (4.42%)	1.14	0.191	0.742247191011236	(A) mut/gain	mut
TCGA	SF3B1	2q33.1	3 (7.32%)	3 (2.65%)	1.46	0.192	0.742247191011236	(A) mut/gain	mut
TCGA	CACNA1D	3p21.1	0 (0.00%)	5 (4.42%)	<-10	0.208	0.742247191011236	(B) mut/neut	mut
TCGA	PTEN	10q23.31	0 (0.00%)	5 (4.42%)	<-10	0.208	0.742247191011236	(B) mut/neut	mut
TCGA	NF1	17q11.2	1 (2.44%)	8 (7.08%)	-1.54	0.255	0.742247191011236	(B) mut/neut	mut
TCGA	EGFR	7p11.2	1 (2.44%)	0 (0.00%)	>10	0.266	0.742247191011236	(A) mut/gain	mut
TCGA	JAK2	9p24.1	1 (2.44%)	0 (0.00%)	>10	0.266	0.742247191011236	(A) mut/gain	mut
TCGA	USP6	17p13.2	1 (2.44%)	0 (0.00%)	>10	0.266	0.742247191011236	(A) mut/gain	mut
TCGA	ARHGEF12	11q23.3	0 (0.00%)	4 (3.54%)	<-10	0.286	0.742247191011236	(B) mut/neut	mut
TCGA	CREBBP	16p13.3	0 (0.00%)	4 (3.54%)	<-10	0.286	0.742247191011236	(B) mut/neut	mut
TCGA	FBXW7	4q31.3	0 (0.00%)	4 (3.54%)	<-10	0.286	0.742247191011236	(B) mut/neut	mut
TCGA	FGFR2	10q26.13	0 (0.00%)	4 (3.54%)	<-10	0.286	0.742247191011236	(B) mut/neut	mut
TCGA	NUMA1	11q13.4	0 (0.00%)	4 (3.54%)	<-10	0.286	0.742247191011236	(B) mut/neut	mut
TCGA	PTPRD	9p24.1-p23	0 (0.00%)	4 (3.54%)	<-10	0.286	0.742247191011236	(B) mut/neut	mut
TCGA	RB1	13q14.2	2 (4.88%)	2 (1.77%)	1.46	0.288	0.742247191011236	(A) mut/gain	mut

TCGA	ZFHX3	16q22.2-q22.3	2 (4.88%)	2 (1.77%)	1.46	0.288	0.742247191011236	(A) mut/gain	mut
TCGA	RUNX1	21q22.12	1 (2.44%)	7 (6.19%)	-1.34	0.321	0.742247191011236	(B) mut/neut	mut
TCGA	PREX2	8q13.2	3 (7.32%)	5 (4.42%)	0.73	0.361	0.742247191011236	(A) mut/gain	mut
TCGA	MAP3K1	5q11.2	6 (14.63%)	21 (18.58%)	-0.34	0.38	0.742247191011236	(B) mut/neut	mut
TCGA	CDKN1B	12p13.1	0 (0.00%)	3 (2.65%)	<-10	0.392	0.742247191011236	(B) mut/neut	mut
TCGA	ESR1	6q25.1-q25.2	0 (0.00%)	3 (2.65%)	<-10	0.392	0.742247191011236	(B) mut/neut	mut
TCGA	SALL4	20q13.2	0 (0.00%)	3 (2.65%)	<-10	0.392	0.742247191011236	(B) mut/neut	mut
TCGA	ARID1A	1p36.11	1 (2.44%)	6 (5.31%)	-1.12	0.401	0.742247191011236	(B) mut/neut	mut
TCGA	CBFB	16q22.1	1 (2.44%)	6 (5.31%)	-1.12	0.401	0.742247191011236	(B) mut/neut	mut
TCGA	GRIN2A	16p13.2	1 (2.44%)	6 (5.31%)	-1.12	0.401	0.742247191011236	(B) mut/neut	mut
TCGA	SPEN	1p36.21-p36.13	1 (2.44%)	6 (5.31%)	-1.12	0.401	0.742247191011236	(B) mut/neut	mut
TCGA	BRCA2	13q13.1	2 (4.88%)	3 (2.65%)	0.88	0.402	0.742247191011236	(A) mut/gain	mut
TCGA	CASP8	2q33.1	2 (4.88%)	3 (2.65%)	0.88	0.402	0.742247191011236	(A) mut/gain	mut
TCGA	KAT6B	10q22.2	2 (4.88%)	3 (2.65%)	0.88	0.402	0.742247191011236	(A) mut/gain	mut
TCGA	MAP2K4	17p12	3 (7.32%)	11 (9.73%)	-0.41	0.46	0.742247191011236	(B) mut/neut	mut
TCGA	CLTC	17q23.1	1 (2.44%)	1 (0.88%)	1.46	0.463	0.742247191011236	(A) mut/gain	mut
TCGA	MYO5A	15q21.2	1 (2.44%)	1 (0.88%)	1.46	0.463	0.742247191011236	(A) mut/gain	mut
TCGA	SMARCD1	12q13.12	1 (2.44%)	1 (0.88%)	1.46	0.463	0.742247191011236	(A) mut/gain	mut
TCGA	FOXA1	14q21.1	2 (4.88%)	4 (3.54%)	0.46	0.507	0.742247191011236	(A) mut/gain	mut
TCGA	ALK	2p23.2-p23.1	0 (0.00%)	2 (1.77%)	<-10	0.537	0.742247191011236	(B) mut/neut	mut
TCGA	ASXL1	20q11.21	0 (0.00%)	2 (1.77%)	<-10	0.537	0.742247191011236	(B) mut/neut	mut
TCGA	CTCF	16q22.1	0 (0.00%)	2 (1.77%)	<-10	0.537	0.742247191011236	(B) mut/neut	mut
TCGA	ELN	7q11.23	0 (0.00%)	2 (1.77%)	<-10	0.537	0.742247191011236	(B) mut/neut	mut
TCGA	EPAS1	2p21	0 (0.00%)	2 (1.77%)	<-10	0.537	0.742247191011236	(B) mut/neut	mut
TCGA	EPHA3	3p11.1	0 (0.00%)	2 (1.77%)	<-10	0.537	0.742247191011236	(B) mut/neut	mut
TCGA	MTOR	1p36.22	0 (0.00%)	2 (1.77%)	<-10	0.537	0.742247191011236	(B) mut/neut	mut
TCGA	PDGFRB	5q32	0 (0.00%)	2 (1.77%)	<-10	0.537	0.742247191011236	(B) mut/neut	mut
TCGA	GATA3	10p14	4 (9.76%)	10 (8.85%)	0.14	0.54	0.742247191011236	(A) mut/gain	mut
TCGA	ARID1B	6q25.3	1 (2.44%)	4 (3.54%)	-0.54	0.598	0.742247191011236	(B) mut/neut	mut
TCGA	ATM	11q22.3	1 (2.44%)	4 (3.54%)	-0.54	0.598	0.742247191011236	(B) mut/neut	mut

TCGA	KRAS	12p12.1	1 (2.44%)	4 (3.54%)	-0.54	0.598	0.742247191011236	(B) mut/neut	mut
TCGA	PTPN13	4q21.3	1 (2.44%)	4 (3.54%)	-0.54	0.598	0.742247191011236	(B) mut/neut	mut
TCGA	FAT4	4q28.1	2 (4.88%)	5 (4.42%)	0.14	0.599	0.742247191011236	(A) mut/gain	mut
TCGA	BAP1	3p21.1	1 (2.44%)	2 (1.77%)	0.46	0.608	0.742247191011236	(A) mut/gain	mut
TCGA	ERBB3	12q13.2	1 (2.44%)	2 (1.77%)	0.46	0.608	0.742247191011236	(A) mut/gain	mut
TCGA	GNAS	20q13.32	1 (2.44%)	2 (1.77%)	0.46	0.608	0.742247191011236	(A) mut/gain	mut
TCGA	MEN1	11q13	1 (2.44%)	2 (1.77%)	0.46	0.608	0.742247191011236	(A) mut/gain	mut
TCGA	NCOA1	2p23.3	1 (2.44%)	2 (1.77%)	0.46	0.608	0.742247191011236	(A) mut/gain	mut
TCGA	PIK3R1	5q13.1	1 (2.44%)	2 (1.77%)	0.46	0.608	0.742247191011236	(A) mut/gain	mut
TCGA	SMAD2	18q21.1	1 (2.44%)	2 (1.77%)	0.46	0.608	0.742247191011236	(A) mut/gain	mut
TCGA	ZBTB16	11q23.2	1 (2.44%)	2 (1.77%)	0.46	0.608	0.742247191011236	(A) mut/gain	mut
TCGA	ABL2	1q25.2	1 (2.44%)	3 (2.65%)	-0.12	0.712	0.742247191011236	(B) mut/neut	mut
TCGA	BRCA1	17q21.31	1 (2.44%)	3 (2.65%)	-0.12	0.712	0.742247191011236	(B) mut/neut	mut
TCGA	ERBB4	2q34	1 (2.44%)	3 (2.65%)	-0.12	0.712	0.742247191011236	(B) mut/neut	mut
TCGA	KDM6A	Xp11.3	1 (2.44%)	3 (2.65%)	-0.12	0.712	0.742247191011236	(B) mut/neut	mut
TCGA	NIN	14q22.1	1 (2.44%)	3 (2.65%)	-0.12	0.712	0.742247191011236	(B) mut/neut	mut
TCGA	RGS7	1q43	1 (2.44%)	3 (2.65%)	-0.12	0.712	0.742247191011236	(B) mut/neut	mut
TCGA	AKT1	14q32.33	0 (0.00%)	1 (0.88%)	<-10	0.734	0.742247191011236	(B) mut/neut	mut
TCGA	BRAF	7q34	0 (0.00%)	1 (0.88%)	<-10	0.734	0.742247191011236	(B) mut/neut	mut
TCGA	DDX3X	Xp11.4	0 (0.00%)	1 (0.88%)	<-10	0.734	0.742247191011236	(B) mut/neut	mut
TCGA	HSP90AA1	14q32.31	0 (0.00%)	1 (0.88%)	<-10	0.734	0.742247191011236	(B) mut/neut	mut
TCGA	KLF4	9q31.2	0 (0.00%)	1 (0.88%)	<-10	0.734	0.742247191011236	(B) mut/neut	mut
TCGA	MAX	14q23.3	0 (0.00%)	1 (0.88%)	<-10	0.734	0.742247191011236	(B) mut/neut	mut
TCGA	MDM4	1q32.1	0 (0.00%)	1 (0.88%)	<-10	0.734	0.742247191011236	(B) mut/neut	mut
TCGA	NCOR2	12q24.31	0 (0.00%)	1 (0.88%)	<-10	0.734	0.742247191011236	(B) mut/neut	mut
TCGA	NONO	Xq13.1	0 (0.00%)	1 (0.88%)	<-10	0.734	0.742247191011236	(B) mut/neut	mut
TCGA	NTRK1	1q23.1	0 (0.00%)	1 (0.88%)	<-10	0.734	0.742247191011236	(B) mut/neut	mut
TCGA	PLAG1	8q12.1	0 (0.00%)	1 (0.88%)	<-10	0.734	0.742247191011236	(B) mut/neut	mut
TCGA	POLD1	19q13.3	0 (0.00%)	1 (0.88%)	<-10	0.734	0.742247191011236	(B) mut/neut	mut
TCGA	RHPN2	19q13.11	0 (0.00%)	1 (0.88%)	<-10	0.734	0.742247191011236	(B) mut/neut	mut

TCGA	ZXDB	Xp11.21	0 (0.00%)	1 (0.88%)	<-10	0.734	0.742247191011236	(B) mut/neut	mut
TCGA	PIK3CA	3q26.32	41 (100.00%)	113 (100.00%)	-	1	1	(B) mut/neut	mut
MSK-meta	CDH1	16q22.1	12 (13.19%)	37 (29.13%)	-1.14	0.003828	0.1893745	(B) mut/neut	mut
MSK-meta	TP53	17p13.1	30 (32.97%)	22 (17.32%)	0.93	0.006209	0.1893745	(A) mut/gain	mut
MSK-meta	ESR1	6q25.1-q25.2	10 (10.99%)	28 (22.05%)	-1.00	0.0245	0.498166666666667	(B) mut/neut	mut
MSK-meta	FAT1	4q35.2	3 (3.30%)	13 (10.24%)	-1.63	0.0432	0.565557142857143	(B) mut/neut	mut
MSK-meta	NCOR1	17p12-p11.2	2 (2.20%)	10 (7.87%)	-1.84	0.0611	0.565557142857143	(B) mut/neut	mut
MSK-meta	CTCF	16q22.1	7 (7.69%)	3 (2.36%)	1.70	0.0643	0.565557142857143	(A) mut/gain	mut
MSK-meta	SMAD4	18q21.2	0 (0.00%)	5 (3.94%)	<-10	0.0649	0.565557142857143	(B) mut/neut	mut
MSK-meta	SPEN	1p36.21-p36.13	7 (7.69%)	4 (3.15%)	1.29	0.116	0.655224137931035	(A) mut/gain	mut
MSK-meta	MAP3K1	5q11.2	16 (17.58%)	14 (11.02%)	0.67	0.118	0.655224137931035	(A) mut/gain	mut
MSK-meta	FOXA1	14q21.1	11 (12.09%)	9 (7.09%)	0.77	0.153	0.655224137931035	(A) mut/gain	mut
MSK-meta	CDKN2A	9p21.3	2 (2.20%)	0 (0.00%)	>10	0.173	0.655224137931035	(A) mut/gain	mut
MSK-meta	EPHA3	3p11.1	2 (2.20%)	0 (0.00%)	>10	0.173	0.655224137931035	(A) mut/gain	mut
MSK-meta	MDM4	1q32.1	2 (2.20%)	0 (0.00%)	>10	0.173	0.655224137931035	(A) mut/gain	mut
MSK-meta	KRAS	12p12.1	0 (0.00%)	3 (2.36%)	<-10	0.196	0.655224137931035	(B) mut/neut	mut
MSK-meta	NTRK1	1q23.1	0 (0.00%)	3 (2.36%)	<-10	0.196	0.655224137931035	(B) mut/neut	mut
MSK-meta	BRCA2	13q13.1	4 (4.40%)	2 (1.57%)	1.48	0.201	0.655224137931035	(A) mut/gain	mut
MSK-meta	BRCA1	17q21.31	1 (1.10%)	5 (3.94%)	-1.84	0.204	0.655224137931035	(B) mut/neut	mut
MSK-meta	FGFR2	10q26.13	1 (1.10%)	5 (3.94%)	-1.84	0.204	0.655224137931035	(B) mut/neut	mut
MSK-meta	GATA3	10p14	15 (16.48%)	16 (12.60%)	0.39	0.268	0.655224137931035	(A) mut/gain	mut
MSK-meta	ERBB2	17q12	2 (2.20%)	6 (4.72%)	-1.10	0.276	0.655224137931035	(B) mut/neut	mut
MSK-meta	KMT2D	12q13.12	6 (6.59%)	5 (3.94%)	0.74	0.282	0.655224137931035	(A) mut/gain	mut
MSK-meta	KDM6A	Xp11.3	1 (1.10%)	4 (3.15%)	-1.52	0.305	0.655224137931035	(B) mut/neut	mut
MSK-meta	MTOR	1p36.22	1 (1.10%)	4 (3.15%)	-1.52	0.305	0.655224137931035	(B) mut/neut	mut
MSK-meta	ARID1A	1p36.11	6 (6.59%)	12 (9.45%)	-0.52	0.31	0.655224137931035	(B) mut/neut	mut
MSK-meta	MEN1	11q13	4 (4.40%)	3 (2.36%)	0.90	0.322	0.655224137931035	(A) mut/gain	mut
MSK-meta	AKT1	14q32.33	3 (3.30%)	2 (1.57%)	1.07	0.347	0.655224137931035	(A) mut/gain	mut
MSK-meta	ERBB3	12q13.2	3 (3.30%)	2 (1.57%)	1.07	0.347	0.655224137931035	(A) mut/gain	mut
MSK-meta	GRIN2A	16p13.2	2 (2.20%)	1 (0.79%)	1.48	0.377	0.655224137931035	(A) mut/gain	mut

MSK-meta	JAK2	9p24.1	2 (2.20%)	1 (0.79%)	1.48	0.377	0.655224137931035	(A) mut/gain	mut
MSK-meta	PDGFRB	5q32	2 (2.20%)	1 (0.79%)	1.48	0.377	0.655224137931035	(A) mut/gain	mut
MSK-meta	NOTCH2	1p12	2 (2.20%)	5 (3.94%)	-0.84	0.38	0.655224137931035	(B) mut/neut	mut
MSK-meta	CBFB	16q22.1	5 (5.49%)	5 (3.94%)	0.48	0.41	0.655224137931035	(A) mut/gain	mut
MSK-meta	NF1	17q11.2	8 (8.79%)	9 (7.09%)	0.31	0.414	0.655224137931035	(A) mut/gain	mut
MSK-meta	BRAF	7q34	1 (1.10%)	0 (0.00%)	>10	0.417	0.655224137931035	(A) mut/gain	mut
MSK-meta	CDKN1B	12p13.1	3 (3.30%)	6 (4.72%)	-0.52	0.437	0.655224137931035	(B) mut/neut	mut
MSK-meta	TBX3	12q24.21	7 (7.69%)	8 (6.30%)	0.29	0.444	0.655224137931035	(A) mut/gain	mut
MSK-meta	ERBB4	2q34	1 (1.10%)	3 (2.36%)	-1.10	0.444	0.655224137931035	(B) mut/neut	mut
MSK-meta	SF3B1	2q33.1	1 (1.10%)	3 (2.36%)	-1.10	0.444	0.655224137931035	(B) mut/neut	mut
MSK-meta	ARID1B	6q25.3	4 (4.40%)	4 (3.15%)	0.48	0.446	0.655224137931035	(A) mut/gain	mut
MSK-meta	KMT2C	7q36.1	13 (14.29%)	17 (13.39%)	0.09	0.5	0.655224137931035	(A) mut/gain	mut
MSK-meta	PTEN	10q23.31	5 (5.49%)	8 (6.30%)	-0.20	0.523	0.655224137931035	(B) mut/neut	mut
MSK-meta	RUNX1	21q22.12	3 (3.30%)	5 (3.94%)	-0.26	0.554	0.655224137931035	(B) mut/neut	mut
MSK-meta	ASXL1	20q11.21	2 (2.20%)	2 (1.57%)	0.48	0.556	0.655224137931035	(A) mut/gain	mut
MSK-meta	FBXW7	4q31.3	2 (2.20%)	2 (1.57%)	0.48	0.556	0.655224137931035	(A) mut/gain	mut
MSK-meta	POLD1	19q13.3	0 (0.00%)	1 (1.09%)	<-10	0.558	0.655224137931035	(B) mut/neut	mut
MSK-meta	ATM	11q22.3	4 (4.40%)	5 (3.94%)	0.16	0.563	0.655224137931035	(A) mut/gain	mut
MSK-meta	CREBBP	16p13.3	4 (4.40%)	5 (3.94%)	0.16	0.563	0.655224137931035	(A) mut/gain	mut
MSK-meta	GATA1	Xp11.23	0 (0.00%)	1 (0.79%)	<-10	0.583	0.655224137931035	(B) mut/neut	mut
MSK-meta	GNAS	20q13.32	0 (0.00%)	1 (0.79%)	<-10	0.583	0.655224137931035	(B) mut/neut	mut
MSK-meta	KLF4	9q31.2	0 (0.00%)	1 (0.79%)	<-10	0.583	0.655224137931035	(B) mut/neut	mut
MSK-meta	SMAD2	18q21.1	0 (0.00%)	1 (0.79%)	<-10	0.583	0.655224137931035	(B) mut/neut	mut
MSK-meta	SMARCD1	12q13.12	0 (0.00%)	1 (0.79%)	<-10	0.583	0.655224137931035	(B) mut/neut	mut
MSK-meta	MAP2K4	17p12	4 (4.40%)	6 (4.72%)	-0.10	0.59	0.655224137931035	(B) mut/neut	mut
MSK-meta	ZFHX3	16q22.2-q22.3	2 (2.74%)	2 (2.17%)	0.33	0.598	0.655224137931035	(A) mut/gain	mut
MSK-meta	RB1	13q14.2	3 (3.30%)	4 (3.15%)	0.07	0.62	0.655224137931035	(A) mut/gain	mut
MSK-meta	BAP1	3p21.1	1 (1.10%)	2 (1.57%)	-0.52	0.623	0.655224137931035	(B) mut/neut	mut
MSK-meta	CASP8	2q33.1	1 (1.10%)	2 (1.57%)	-0.52	0.623	0.655224137931035	(B) mut/neut	mut
MSK-meta	PTPRD	9p24.1-p23	1 (1.10%)	2 (1.57%)	-0.52	0.623	0.655224137931035	(B) mut/neut	mut

MSK-meta	ALK	2p23.2-p23.1	1 (1.10%)	1 (0.79%)	0.48	0.662	0.673033333333333	(A) mut/gain	mut
MSK-meta	EGFR	7p11.2	1 (1.10%)	1 (0.79%)	0.48	0.662	0.673033333333333	(A) mut/gain	mut
MSK-meta	PIK3CA	3q26.32	91 (100.00%)	127 (100.00%)	-	1	1	(B) mut/neut	mut
METABRIC	TP53	17p13.1	44 (38.26%)	65 (12.55%)	1.61	1.03e-09	4,43E-05	(A) mut/gain	mut
METABRIC	SF3B1	2q33.1	13 (11.30%)	18 (3.47%)	1.70	0.001356	0.029154	(A) mut/gain	mut
METABRIC	GATA3	10p14	7 (6.09%)	82 (15.83%)	-1.38	0.002985	0.042785	(B) mut/neut	mut
METABRIC	MAP2K4	17p12	1 (0.87%)	28 (5.41%)	-2.64	0.0201	0.1892	(B) mut/neut	mut
METABRIC	CDH1	16q22.1	9 (7.83%)	79 (15.25%)	-0.96	0.022	0.1892	(B) mut/neut	mut
METABRIC	SMAD4	18q21.2	5 (4.35%)	6 (1.16%)	1.91	0.0333	0.213157142857143	(A) mut/gain	mut
METABRIC	TBX3	12q24.21	3 (2.61%)	39 (7.53%)	-1.53	0.0347	0.213157142857143	(B) mut/neut	mut
METABRIC	BRCA1	17q21.31	5 (4.35%)	7 (1.35%)	1.69	0.049	0.215781818181818	(A) mut/gain	mut
METABRIC	BRCA2	13q13.1	5 (4.35%)	7 (1.35%)	1.69	0.049	0.215781818181818	(A) mut/gain	mut
METABRIC	KMT2C	7q36.1	16 (13.91%)	109 (21.04%)	-0.60	0.0506	0.215781818181818	(B) mut/neut	mut
METABRIC	CBFB	16q22.1	5 (4.35%)	48 (9.27%)	-1.09	0.0552	0.215781818181818	(B) mut/neut	mut
METABRIC	CDKN1B	12p13.1	0 (0.00%)	12 (2.32%)	<-10	0.0881	0.315691666666667	(B) mut/neut	mut
METABRIC	NF1	17q11.2	3 (2.61%)	30 (5.79%)	-1.15	0.119	0.393615384615385	(B) mut/neut	mut
METABRIC	ARID1B	6q25.3	6 (5.22%)	14 (2.70%)	0.95	0.137	0.4111875	(A) mut/gain	mut
METABRIC	ERBB2	17q12	5 (4.35%)	11 (2.12%)	1.03	0.147	0.4111875	(A) mut/gain	mut
METABRIC	SMAD2	18q21.1	2 (1.74%)	2 (0.39%)	2.17	0.153	0.4111875	(A) mut/gain	mut
METABRIC	ALK	2p23.2-p23.1	5 (4.35%)	12 (2.32%)	0.91	0.18	0.449111111111111	(A) mut/gain	mut
METABRIC	NCOR1	17p12-p11.2	9 (7.83%)	27 (5.21%)	0.59	0.188	0.449111111111111	(A) mut/gain	mut
METABRIC	RB1	13q14.2	0 (0.00%)	8 (1.54%)	<-10	0.199	0.450368421052632	(B) mut/neut	mut
METABRIC	MYH9	22q12.3	5 (4.35%)	13 (2.51%)	0.79	0.215	0.46225	(A) mut/gain	mut
METABRIC	CTCF	16q22.1	2 (1.74%)	19 (3.67%)	-1.08	0.233	0.476909090909091	(B) mut/neut	mut
METABRIC	EGFR	7p11.2	0 (0.00%)	7 (1.35%)	<-10	0.244	0.476909090909091	(B) mut/neut	mut
METABRIC	ARID1A	1p36.11	5 (4.35%)	33 (6.37%)	-0.55	0.28	0.523478260869565	(B) mut/neut	mut
METABRIC	PTEN	10q23.31	3 (2.61%)	22 (4.25%)	-0.70	0.305	0.546458333333333	(B) mut/neut	mut
METABRIC	NCOR2	12q24.31	6 (5.22%)	21 (4.05%)	0.36	0.364	0.55751724137931	(A) mut/gain	mut
METABRIC	AKT1	14q32.33	0 (0.00%)	5 (0.97%)	<-10	0.366	0.55751724137931	(B) mut/neut	mut
METABRIC	BAP1	3p21.1	2 (1.74%)	5 (0.97%)	0.85	0.373	0.55751724137931	(A) mut/gain	mut

METABRIC	PIK3R1	5q13.1	2 (1.74%)	5 (0.97%)	0.85	0.373	0.55751724137931	(A) mut/gain	mut
METABRIC	KMT2D	12q13.12	9 (7.83%)	34 (6.56%)	0.25	0.376	0.55751724137931	(A) mut/gain	mut
METABRIC	ERBB4	2q34	1 (0.87%)	9 (1.74%)	-1.00	0.432	0.6192	(B) mut/neut	mut
METABRIC	MAP3K1	5q11.2	22 (19.13%)	95 (18.34%)	0.06	0.467	0.628875	(A) mut/gain	mut
METABRIC	KDM6A	Xp11.3	2 (1.74%)	13 (2.51%)	-0.53	0.468	0.628875	(B) mut/neut	mut
METABRIC	CASP8	2q33.1	2 (1.74%)	7 (1.35%)	0.36	0.508	0.640589743589744	(A) mut/gain	mut
METABRIC	CDKN2A	9p21.3	0 (0.00%)	3 (0.58%)	<-10	0.547	0.640589743589744	(B) mut/neut	mut
METABRIC	MEN1	11q13	1 (0.87%)	3 (0.58%)	0.59	0.553	0.640589743589744	(A) mut/gain	mut
METABRIC	RUNX1	21q22.12	5 (4.35%)	24 (4.63%)	-0.09	0.564	0.640589743589744	(B) mut/neut	mut
METABRIC	ERBB3	12q13.2	3 (2.61%)	13 (2.51%)	0.06	0.579	0.640589743589744	(A) mut/gain	mut
METABRIC	ASXL1	20q11.21	3 (2.61%)	15 (2.90%)	-0.15	0.581	0.640589743589744	(B) mut/neut	mut
METABRIC	PTPRD	9p24.1-p23	3 (2.61%)	15 (2.90%)	-0.15	0.581	0.640589743589744	(B) mut/neut	mut
METABRIC	FBXW7	4q31.3	1 (0.87%)	4 (0.77%)	0.17	0.634	0.68155	(A) mut/gain	mut
METABRIC	BRAF	7q34	0 (0.00%)	2 (0.39%)	<-10	0.669	0.684928571428571	(B) mut/neut	mut
METABRIC	KRAS	12p12.1	0 (0.00%)	2 (0.39%)	<-10	0.669	0.684928571428571	(B) mut/neut	mut
METABRIC	PIK3CA	3q26.32	115 (100.00%)	518 (100.00%)	-	1	1	(B) mut/neut	mut
MSK2018	TP53	17p13.1	16 (42.11%)	20 (10.36%)	2.02	1,07E-02	0.00066154	(A) mut/gain	mut
MSK2018	SF3B1	2q33.1	4 (10.53%)	3 (1.55%)	2.76	0.0153	0.4743	(A) mut/gain	mut
MSK2018	CDH1	16q22.1	5 (13.16%)	54 (27.98%)	-1.09	0.0384	0.7936	(B) mut/neut	mut
MSK2018	CBFB	16q22.1	1 (2.63%)	24 (12.44%)	-2.24	0.0561	0.838192307692308	(B) mut/neut	mut
MSK2018	GATA3	10p14	2 (5.26%)	27 (13.99%)	-1.41	0.106	0.838192307692308	(B) mut/neut	mut
MSK2018	CDKN2A	9p21.3	2 (5.26%)	2 (1.04%)	2.34	0.127	0.838192307692308	(A) mut/gain	mut
MSK2018	GRIN2A	16p13.2	2 (5.26%)	2 (1.04%)	2.34	0.127	0.838192307692308	(A) mut/gain	mut
MSK2018	RB1	13q14.2	2 (5.26%)	2 (1.04%)	2.34	0.127	0.838192307692308	(A) mut/gain	mut
MSK2018	RUNX1	21q22.12	4 (10.53%)	9 (4.66%)	1.17	0.147	0.838192307692308	(A) mut/gain	mut
MSK2018	EPAS1	2p21	1 (4.76%)	0 (0.00%)	>10	0.149	0.838192307692308	(A) mut/gain	mut
MSK2018	HRAS	11p15.5	1 (2.63%)	0 (0.00%)	>10	0.165	0.838192307692308	(A) mut/gain	mut
MSK2018	NTRK1	1q23.1	1 (2.63%)	0 (0.00%)	>10	0.165	0.838192307692308	(A) mut/gain	mut
MSK2018	PTEN	10q23.31	0 (0.00%)	9 (4.66%)	<-10	0.192	0.838192307692308	(B) mut/neut	mut
MSK2018	BRCA2	13q13.1	0 (0.00%)	7 (3.63%)	<-10	0.279	0.838192307692308	(B) mut/neut	mut

MSK2018	BAP1	3p21.1	1 (2.63%)	1 (0.52%)	2.34	0.303	0.838192307692308	(A) mut/gain	mut
MSK2018	MAP3K1	5q11.2	4 (10.53%)	29 (15.03%)	-0.51	0.331	0.838192307692308	(B) mut/neut	mut
MSK2018	FOXA1	14q21.1	1 (2.63%)	12 (6.22%)	-1.24	0.336	0.838192307692308	(B) mut/neut	mut
MSK2018	KMT2C	7q36.1	2 (5.26%)	17 (8.81%)	-0.74	0.364	0.838192307692308	(B) mut/neut	mut
MSK2018	FAT1	4q35.2	2 (5.26%)	6 (3.11%)	0.76	0.39	0.838192307692308	(A) mut/gain	mut
MSK2018	ATM	11q22.3	0 (0.00%)	5 (2.59%)	<-10	0.404	0.838192307692308	(B) mut/neut	mut
MSK2018	CTCF	16q22.1	0 (0.00%)	5 (2.59%)	<-10	0.404	0.838192307692308	(B) mut/neut	mut
MSK2018	ERBB3	12q13.2	0 (0.00%)	5 (2.59%)	<-10	0.404	0.838192307692308	(B) mut/neut	mut
MSK2018	ESR1	6q25.1-q25.2	0 (0.00%)	5 (2.59%)	<-10	0.404	0.838192307692308	(B) mut/neut	mut
MSK2018	ZFHX3	16q22.2-q22.3	0 (0.00%)	5 (2.91%)	<-10	0.412	0.838192307692308	(B) mut/neut	mut
MSK2018	KMT2D	12q13.12	0 (0.00%)	4 (2.07%)	<-10	0.485	0.838192307692308	(B) mut/neut	mut
MSK2018	NF1	17q11.2	0 (0.00%)	4 (2.07%)	<-10	0.485	0.838192307692308	(B) mut/neut	mut
MSK2018	NOTCH2	1p12	0 (0.00%)	4 (2.07%)	<-10	0.485	0.838192307692308	(B) mut/neut	mut
MSK2018	SPEN	1p36.21-p36.13	1 (2.63%)	9 (4.66%)	-0.83	0.488	0.838192307692308	(B) mut/neut	mut
MSK2018	NCOR1	17p12-p11.2	3 (7.89%)	13 (6.74%)	0.23	0.508	0.838192307692308	(A) mut/gain	mut
MSK2018	TBX3	12q24.21	3 (7.89%)	13 (6.74%)	0.23	0.508	0.838192307692308	(A) mut/gain	mut
MSK2018	MAP2K4	17p12	2 (5.26%)	8 (4.15%)	0.34	0.512	0.838192307692308	(A) mut/gain	mut
MSK2018	ERBB2	17q12	1 (2.63%)	3 (1.55%)	0.76	0.515	0.838192307692308	(A) mut/gain	mut
MSK2018	KLF4	9q31.2	1 (2.63%)	3 (1.55%)	0.76	0.515	0.838192307692308	(A) mut/gain	mut
MSK2018	MEN1	11q13	1 (2.63%)	3 (1.55%)	0.76	0.515	0.838192307692308	(A) mut/gain	mut
MSK2018	SMAD4	18q21.2	1 (2.63%)	3 (1.55%)	0.76	0.515	0.838192307692308	(A) mut/gain	mut
MSK2018	CDKN1B	12p13.1	1 (2.63%)	8 (4.15%)	-0.66	0.548	0.838192307692308	(B) mut/neut	mut
MSK2018	ALK	2p23.2-p23.1	0 (0.00%)	3 (1.55%)	<-10	0.582	0.838192307692308	(B) mut/neut	mut
MSK2018	EGFR	7p11.2	0 (0.00%)	3 (1.55%)	<-10	0.582	0.838192307692308	(B) mut/neut	mut
MSK2018	KDM6A	Xp11.3	0 (0.00%)	3 (1.55%)	<-10	0.582	0.838192307692308	(B) mut/neut	mut
MSK2018	KRAS	12p12.1	0 (0.00%)	3 (1.55%)	<-10	0.582	0.838192307692308	(B) mut/neut	mut
MSK2018	PTPRD	9p24.1-p23	0 (0.00%)	3 (1.55%)	<-10	0.582	0.838192307692308	(B) mut/neut	mut
MSK2018	BRAF	7q34	1 (2.63%)	4 (2.07%)	0.34	0.596	0.838192307692308	(A) mut/gain	mut
MSK2018	MTOR	1p36.22	1 (2.63%)	4 (2.07%)	0.34	0.596	0.838192307692308	(A) mut/gain	mut
MSK2018	ARID1A	1p36.11	2 (5.26%)	10 (5.18%)	0.02	0.617	0.838192307692308	(A) mut/gain	mut

MSK2018	AKT1	14q32.33	0 (0.00%)	2 (1.04%)	<-10	0.697	0.838192307692308	(B) mut/neut	mut
MSK2018	ASXL1	20q11.21	0 (0.00%)	2 (1.04%)	<-10	0.697	0.838192307692308	(B) mut/neut	mut
MSK2018	BRCA1	17q21.31	0 (0.00%)	2 (1.04%)	<-10	0.697	0.838192307692308	(B) mut/neut	mut
MSK2018	CREBBP	16p13.3	0 (0.00%)	2 (1.04%)	<-10	0.697	0.838192307692308	(B) mut/neut	mut
MSK2018	EPHA3	3p11.1	0 (0.00%)	2 (1.04%)	<-10	0.697	0.838192307692308	(B) mut/neut	mut
MSK2018	GNAS	20q13.32	0 (0.00%)	2 (1.04%)	<-10	0.697	0.838192307692308	(B) mut/neut	mut
MSK2018	MAX	14q23.3	0 (0.00%)	2 (1.04%)	<-10	0.697	0.838192307692308	(B) mut/neut	mut
MSK2018	POLD1	19q13.3	0 (0.00%)	2 (1.16%)	<-10	0.703	0.838192307692308	(B) mut/neut	mut
MSK2018	PREX2	8q13.2	0 (0.00%)	2 (1.67%)	<-10	0.723	0.84577358490566	(B) mut/neut	mut
MSK2018	ARID1B	6q25.3	0 (0.00%)	1 (0.52%)	<-10	0.835	0.848688524590164	(B) mut/neut	mut
MSK2018	ERBB4	2q34	0 (0.00%)	1 (0.52%)	<-10	0.835	0.848688524590164	(B) mut/neut	mut
MSK2018	FBXW7	4q31.3	0 (0.00%)	1 (0.52%)	<-10	0.835	0.848688524590164	(B) mut/neut	mut
MSK2018	GATA1	Xp11.23	0 (0.00%)	1 (0.52%)	<-10	0.835	0.848688524590164	(B) mut/neut	mut
MSK2018	JAK2	9p24.1	0 (0.00%)	1 (0.52%)	<-10	0.835	0.848688524590164	(B) mut/neut	mut
MSK2018	PDGFRB	5q32	0 (0.00%)	1 (0.52%)	<-10	0.835	0.848688524590164	(B) mut/neut	mut
MSK2018	SMAD2	18q21.1	0 (0.00%)	1 (0.52%)	<-10	0.835	0.848688524590164	(B) mut/neut	mut
MSK2018	SMARCD1	12q13.12	0 (0.00%)	1 (0.52%)	<-10	0.835	0.848688524590164	(B) mut/neut	mut
MSK2018	PIK3CA	3q26.32	38 (100.00%)	193 (100.00%)	-	1	1	(B) mut/neut	mut
